# Insights into the mechanism of Huanglongbing tolerance in the Australian finger lime (*Citrus australasica*)

**DOI:** 10.3389/fpls.2022.1019295

**Published:** 2022-10-21

**Authors:** Kyle C. Weber, Lamiaa M. Mahmoud, Daniel Stanton, Stacy Welker, Wenming Qiu, Jude W. Grosser, Amit Levy, Manjul Dutt

**Affiliations:** ^1^ Citrus Research and Education Center, University of Florida, Lake Alfred, FL, United States; ^2^ Pomology Department, Faculty of Agriculture, Mansoura University, Mansoura, Egypt; ^3^ Institute of Fruit and Tea, Hubei Academy of Agricultural Sciences, Wuhan, China

**Keywords:** citrus, transcriptome, Huanglongbing, host response, pathogen-related proteins, callose deposition

## Abstract

The Australian finger lime (*Citrus australasica*) is tolerant to Huanglongbing (HLB; Citrus greening). This species can be utilized to develop HLB tolerant citrus cultivars through conventional breeding and biotechnological approaches. In this report, we conducted a comprehensive analysis of transcriptomic data following a non-choice infection assay to understand the *Ca*Las tolerance mechanisms in the finger lime. After filtering 3,768 differentially expressed genes (DEGs), 2,396 were downregulated and 1,372 were upregulated in *Ca*Las-infected finger lime compared to *Ca*Las-infected HLB-susceptible ‘Valencia’ sweet orange. Comparative analyses revealed several DEGs belonging to cell wall, β-glucanase, proteolysis, R genes, signaling, redox state, peroxidases, glutathione-S-transferase, secondary metabolites, and pathogenesis-related (PR) proteins categories. Our results indicate that the finger lime has evolved specific redox control systems to mitigate the reactive oxygen species and modulate the plant defense response. We also identified candidate genes responsible for the production of Cys-rich secretory proteins and Pathogenesis-related 1 (PR1-like) proteins that are highly upregulated in infected finger lime relative to noninfected and infected ‘Valencia’ sweet orange. Additionally, the anatomical analysis of phloem and stem tissues in finger lime and ‘Valencia’ suggested better regeneration of phloem tissues in finger lime in response to HLB infection. Analysis of callose formation following infection revealed a significant difference in the production of callose plugs between the stem phloem of *Ca*Las+ ‘Valencia’ sweet orange and finger lime. Understanding the mechanism of resistance will help the scientific community design strategies to protect trees from *Ca*Las infection and assist citrus breeders in developing durable HLB tolerant citrus varieties.

## Introduction

The genus Citrus originated in tropical and subtropical southeastern Asia (Wu et al., 2014). When consumed fresh, citrus fruit are good sources of dietary fiber (Marín et al., 2007) and antioxidants (Wang et al., 2022), and they have anticancer and anti-inflammatory properties (Benavente-García and Castillo, 2008). The United States is one of the citrus producers, with production concentrated in Florida, California, and Texas. Sweet orange constitutes most of the citrus production acreage, with the remainder being that of grapefruit, mandarin, lemons and limes (Kramer et al., 2022).

Citrus is susceptible to plethora of diseases and pests, with huanglongbing (HLB), a phloem-limiting bacterial disease caused by the bacterium *Candidatus* Liberibacter asiaticus (*Ca*Las), being the most destructive (Duan et al., 2009). In the United States, this disease has been prevalent since 2005 (Bové, 2006), when it was first detected in south Florida’s Miami-Dade County (Halbert, 2005). Widespread monoculture of a few select citrus varieties has reduced the genetic diversity of cultivated citrus, allowing HLB to spread quickly among the population. Since its initial discovery in 2005, HLB has spread rapidly throughout Florida to every citrus-growing county. Additionally, HLB is now present in Texas and California, where it threatens the important central valley (Milne et al., 2018; Graham et al., 2020). Most of the commercial citrus cultivars grown in the United States, including several named cultivars and selections of sweet orange, mandarin, and grapefruit, are highly susceptible to HLB.

The health of infected trees invariably declines, accompanied by reduced fruit yield and quality (Ferguson et al., 2021) and severely infected trees eventually die (Zhang et al., 2020b). Long-term management of tree health through enhanced nutrition (Zambon et al., 2019) and psyllid vector control using various control strategies have been proposed and evaluated (Mattos-Jr et al., 2020). Tolerance to HLB has been reported in some citrus cultivars, such as citron and its hybrids (e.g., lemons), and in some trifoliate orange trees and their hybrids (Peng et al., 2020). HLB-tolerant scions and rootstocks, conventionally bred or transgenic, remain the best option for the control and management of HLB (Qiu et al., 2020). Sugar Belle, a recently released mandarin hybrid, has also been observed to be HLB tolerant (Killiny et al., 2018). Several wild sexually compatible cultivars, such as *Citrus ichangensis* ‘2586’ (Wu et al., 2020), *Citrus latipes* (Folimonova et al., 2009), several accessions of sour pummelo (*Citrus grandis;* Zou et al. (2019) and kaffir lime (*Citrus hystrix;* Hu et al. (2017), are also tolerant to HLB. Additionally, several sexually incompatible citrus relatives are also tolerant to HLB (Miles et al., 2017).

The Australian limes spread from Southeast Asia to Australasia during the early Pliocene epoch, approximately 4 Ma (Wu et al., 2018). There are seven species of Australian limes that are all native to Australia and the New Guinea islands (Forster and Smith, 2010). Australian lime species such as *Citrus australasica*, and the hybrid of *Citrus australis* and *Citrus virgata* (Sydney hybrid) have been reported to be HLB resistant (Ramadugu et al., 2016; Alves et al., 2020; Huang et al., 2021a). Thus, these species can provide pathogen resistance-related genes that can be used to confer HLB tolerance into conventional citrus cultivars to produce HLB-tolerant citrus hybrid scions and rootstocks. Although extensive research is being conducted to use HLB tolerance traits for the development of new citrus cultivars, there is a lack of knowledge on the mechanism underlying the perceived tolerance in these citrus species. Resistance and tolerance observed in the different cultivars can be defined by various factors, including the absence of *Ca*Las multiplication and replication, delayed infection, or recovery from infection by enhancing plant defensive systems.

*Ca*Las is a gram-negative bacterium that employs secretion systems that deliver virulence proteins, known as effectors, to manipulate its hosts (Clark et al., 2018) through modulation of host physiology and suppressing plant defense mechanisms. Effectors promote pathogen colonization and disease development and create environmental conditions favorable for colonization and proliferation (Jones and Dangl, 2006; Dou and Zhou, 2012; Feng and Zhou, 2012). The plant defense response system involves pattern-triggered immunity (PTI), which is triggered by microbe-associated molecular patterns (MAMPs) *via* cell surface-localized pattern-recognition receptors (PRRs), and effector-triggered immunity (ETI), which is induced by pathogen effector proteins *via* intracellular receptors that detect intercellular pathogen-derived molecules and intracellular receptors that activate plant defense response upon detection of pathogen-secreted effector proteins that function inside the plant cell (Ngou et al., 2021). Previous research showed that *Ca*Las encodes several Sec-delivered effectors (SDEs), many of which are conserved across *Ca*Las isolates. Sec-delivered effector 1 (SDE1), a secreted protein biomarker used for the detection of HLB, is highly expressed in infected citrus tissue at a relatively early infection stage (Pagliaccia et al., 2017; Tran et al., 2020).

Proteases secreted by pathogens have been shown to be important virulence factors that affect plant defense, and cysteine (Cys) proteases have been demonstrated to participate in different pathosystems (Zhang et al., 2020a). Citrus papain-like cysteine proteases (PLCPs) were found as a defense inducible in *Ca*Las-infected trees, suggesting they are involved in the citrus defense responses (Clark et al., 2018). Additionally, several lysosomal Cys proteases were shown to be involved in various apoptosis models, although the mechanisms of their involvement are not yet clear (Rozman-Pungerčar et al., 2003). Plasma membrane-localized receptor-like kinases (RLKs) play a role in plant recognition of microbes and in perceiving and transducing these external stimuli to further activate the associated downstream signaling pathways (Jose et al., 2020). RLKs are categorized into several subfamilies, including leucine-rich repeat (LRR) RLKs (LRR-RLKs), Cys-rich repeat (CRKs), domains of unknown function 26 RLKs, S-domain RLKs, and others (Quezada et al., 2019).

In this study, we shed light on the potential mechanism of HLB tolerance in the finger lime. To this end, we graft-inoculated one-year-mature finger lime and ‘Valencia’ (*Citrus sinensis*) sweet orange trees with *Ca*Las and evaluated their transcriptome to provide insights into the mechanism of tolerance to HLB. The transcriptome data was also validated in five-year-mature trees growing in the field.

## Materials and methods

### Plant materials

Certified HLB-free budwood of *C. australasica* clone DPI 50-36 (Finger lime) and ‘Valencia’ sweet orange clone SPB-1-14-19 were obtained from Florida’s Division of Plant Industry budwood repository and budded onto 6-month-old Swingle citrumelo rootstock. One-year-old budded trees were subsequently side grafted (**Figure S1**) with *Ca*Las-infected ‘Valencia’ sweet orange scions (Ct value of 23.2 ± 0.3). The trees were periodically evaluated for infection, and 1 to 2 year-old infected trees were utilized for subsequent experiments (**Table S1**).

### Monitoring *Ca*Las in finger lime and ‘valencia’ sweet orange plants

To diagnose the *Ca*Las titer in the potentially infected greenhouse-grown trees, genomic DNA was isolated periodically from the leaf petioles and midveins of fully expanded leaves using a GeneJET Plant Genomic DNA Purification Kit (Thermo Fisher Scientific Waltham, MA, USA). Leaves were also collected in the late fall (November) and early spring (March) from 5-year-old finger lime DPI 50-36 and ‘Valencia’ SPB-1-14-19 trees growing in the field (Swingle rootstock) to estimate the *Ca*Las titer in the sampled tissues for three years. The DNA concentration was normalized to 25 ng/μL before performing qPCR using a StepOnePlus™ Real-Time PCR System (Thermo Fisher Scientific). Detection of *Ca*Las genomic DNA was determined by qPCR using TaqMan™ Gene Expression Master Mix and CQUL primers (**Table S2**) to amplify the *Ca*Las rplJ/rplL ribosomal protein gene (Wang et al., 2006).

### RNA extraction, cDNA synthesis and sequencing

Two years following infection, RNA was extracted using TRIzol^®^ following the manufacturer’s protocol. The purity and integrity of the RNA were analyzed using electrophoresis on a 1.0% agarose gel and then examined using an Agilent 2100 Bioanalyzer (Agilent Technologies, Santa Clara, CA, USA). High-quality RNA samples with an RNA integrity number (RIN) > 6.5 were used for cDNA synthesis and RNA sequencing (RNAseq). Single-stranded cDNA was synthesized using a RevertAid First Strand cDNA Synthesis Kit (Thermo Fisher Scientific, Massachusetts, USA). The cDNA concentration was determined using a NanoDrop™ 1000 Spectrophotometer (Thermo Fisher Scientific).

The cDNA libraries were sequenced using an Illumina HiSeq platform configured for a 2x150 read length. The generated base callings of the cDNA reads were presented in a paired-ended format. The reads were cleaned, and their adapters were removed using AdapterRemoval v2.2.2 (Lindgreen, 2012), with the default parameters. Short and poor-quality reads were filtered using Trimmomatic v0.39 (Bolger et al., 2014). The following parameters were applied: a minimum length of 100 bases, a trailing and leading length equal to 16 bases, a sliding window of 16:25, and 5 threads. After processing, the final read count and average qualities were checked using FastQC v0.11.8 (Andrews, 2010).

### Mapping of the reads, transcript counts, and DEG analysis

The cleaned reads were mapped to the *C. sinensis* genome using STAR v2.6.0C (Dobin et al., 2013) with the default parameters, except for the need to define a sorted BAM output. The *C. sinensis* genome and annotations used in STAR were obtained from Phytozome (https://phytozome-next.jgi.doe.gov/) The BAM files were indexed using SAMtools v1.7 (Li et al., 2009). The BAM files were assessed for transcript counts using featureCounts v1.6.0 (Liao et al., 2014) with default settings except for the section of exon type and five threads. The list of counts was extracted from the featureCounts software output file and organized to compare infected finger lime *vs*. infected ‘Valencia’ sweet orange. A metadata file for the comparison was also generated for differentially expressed gene (DEG) analysis *via* DESeq2 v3.10, an R Bioconductor package (Love et al., 2014). The counts for each comparison were normalized, and gene dispersion was estimated using DESeq2. The list of DEGs was filtered to remove any DEGs with a |log_2_(fold-change)|< 2 and an adjusted *P* value (false discovery rate (FDR)) ≥ 0.05.

### Gene ontology enrichment and pathway analysis

Statistically significant DEGs were analyzed using AgriGO v2 (Tian et al., 2017). To correctly assign GO terms, the following parameters were selected: Genome - *Citrus sinensis*, statistical test - Fisher’s exact test, adjusted according to the Benjamini–Yekutieli method (Benjamini et al., 2006) for discovering FDRs in a multiple comparison with an alpha=0.05, and a minimum mapping of 5. The statistically significant GO terms from AgriGO v2 were then inputted into REVIGO software to remove redundant GO terms (Supek et al., 2011). Functional analysis was conducted using the *C. sinensis* pathways file (m02) in MapMan (Thimm et al., 2004). The functional categories were viewed using PageMan and analyzed for statistical significance using a nonparametric test (Usadel et al., 2006). Pathway analysis was performed using the pathways function of MapMan (Mapman version 3.0.0).

### Quantitative PCR and DEG validation

The real-time PCR (qPCR) reaction mix consisted of 1 µL of DNA (25 ng/µL), SYBR^®^ Green PowerUp™ PCR Master Mix (Applied Biosystems, Foster City, CA), and selected gene-specific primers (Integrated DNA Technologies, Inc., Coralville, IA, USA) in a final mixture of 20 μL, according to the manufacturers’ instructions. qPCR was performed in a StepOnePlus™ Real-Time PCR System (Thermo Fisher Scientific, Massachusetts, USA). The citrus β-actin housekeeping gene was used as a reference gene (Qiu et al., 2020); each sample was analyzed in triplicate. Relative gene expression was calculated using the 2^-ΔΔCt^ method described previously (Livak and Schmittgen, 2001). The relative mRNA levels were compared to those of the endogenous *C. sinensis* ACTIN gene (Qiu et al., 2020) and calculated using the 2^-ΔΔCT^ method (Livak and Schmittgen, 2001). To confirm the validity of the DEGs, we selected eight upregulated and eight downregulated genes from the DEG data, then analyzed on the same samples that were sequenced and the relative expression of those genes were compared with RNAseq results. As there is no publicly available genome assembly of the finger lime yet, we selected those genes based on the sweet orange genome. Additionally, some of the DEGs that show significant difference were validated in twelve samples collected from finger lime and ‘Valencia’ sweet orange trees growing in the field. The gene expression of the trees was compared with that of uninfected control trees growing in a protected greenhouse. The control trees were confirmed to be negative for *Ca*Las before subsequent comparison. A list of the primers used in this study is presented in **Tables S3, S4**.

### Proteolytic enzyme assays

Assessment of Cys protease activity was performed by recording the liberation of fluorogenic peptide substrate VIII (Z-Phe-Arg-AMC. Z: N-carbobenzyloxy; 7-amino-4-methylcoumarin; Z-F-R-AMC), given that protease activity correlates with an increase in detectable relative light units (RFUs) over time (Barrett, 1980; Tchoupé et al., 1991). The leaf samples were extracted in a buffer consisting of 100 mM sodium acetate (pH 5.5), 2.5 mM DTT and 1 mM EDTA. The samples were centrifuged, and the supernatants were incubated at a 1:2 ratio together with a mixture consisting of 100 mM sodium acetate (pH 5.5), 2.5 mM DTT, 1 mM EDTA, 0.5% DMSO, and 37.5 mM Z-F-R-AMC. All the samples were incubated at 30°C for 5 minutes. Another set of samples were co-incubated for 3 hours with 10 µM synthetic epoxide peptide E-64 (L-3-trans-carboxyoxiran-2-carbonyl]-L-Leu-agmatin]; [N-(transepoxysuccinyl)-L-leucine 4-guanidinobutylamide]) as inhibitor of cysteine protease at 37°C. At the end of the incubation, fluorescence was measured in a Thermo Scientific™ GENESYS™ 30 Visible Spectrophotometer at λex = 380 and λem = 460 nm. Negative (no enzyme) and blank samples were also prepared along with the positive samples by the addition of E-64 inhibitor and solvent, respectively. The percent inhibition was calculated by using the following formula:

Inhibition % = [Absorbance (blank)-Absorbance (test)]/Absorbance (blank) x 100

### Evaluation of *Ca*Las*-*infected ‘valencia’ sweet orange and finger lime and quantification of phloem callose deposits

Healthy and *Ca*Las-infected finger lime and ‘Valencia’ sweet orange leaves (position 5^th^ -8^th^ from the apical meristem) were collected from the greenhouse. The petioles were cut and fixed in 4% paraformaldehyde in 1x PBS. The samples were rinsed three times in 1x PBS and then dehydrated in an ethanol (EtOH) series for 1 hour each. The samples were transitioned from 100% EtOH to 100% tert-butanol (3:1, 1:1, and 1:3) at room temperature (RT) for 8-16 hours each and then cleared in 100% tert-butanol for one hour prior to paraffin infiltration. The samples were infiltrated using increasing concentrations (3:1, 1:1, 1:3) of Paraplast Plus paraffin (Fisher Scientific, Waltham, MA, USA) for 24 hours each and then incubated for 48-36 hours in 100% paraffin, which was changed three times. The samples were embedded in paraffin and allowed to harden for 24 hours at 4°C. Afterward, ten micrometer sections were cut using a Leica 2155 microtome (Leica Biosystems, Deer Park IL, USA), and the sections were floated on a drop of water on a slide. The slides were subsequently incubated overnight on a slide warmer at 37°C to allow the sections to adhere to the slide. The slides were dewaxed in 100% Histoclear II (National Diagnostics, Atlanta, GA, USA) for an hour each, and the solution was changed twice. The sections were stained with 0.05% toluidine blue O for 30 seconds and then rinsed in dH_2_O. The slides were dehydrated in an EtOH series for 5-10 minutes each. Coverslips were mounted using Fisher Scientific’s mounting media with toluene (Fisher Scientific, Waltham, MA, USA). The slides were observed under an Olympus BX61 epifluorescence microscope (Olympus, Center Valley, PA, USA), and images were captured using a 14 MP OMAX digital camera (OMAX, Irvine, CA). The phloem and xylem ring distances were measured using FIJI (Schindelin et al., 2012) **Figure S2**, and the phloem ratio (Pa/Xa) and xylem ratio (Xa/Pa) for petioles and stems were calculated. Three samples were used for evaluation and the data were recorded as average for three images of each sample.

Phloem sieve plate callose was measured according to a previous protocol (Ferrara et al., 2015). Stem phloem tissue samples were collected from *Ca*Las+ ‘Valencia’ sweet orange and finger lime trees. The tissue samples were obtained from the stems of the mature trees with a scalpel, approximately 8 cm from the leaves. Three stem phloem samples were collected from five trees of each plant type. Each tissue sample was placed into an 85% EtOH solution for fixation immediately after collection and incubated overnight for de-staining. The samples were then transferred to a 0.01% Tween-20 solution to rehydrate for 1 hour. Finally, the samples were transferred to a 0.01% aniline blue staining solution. After staining for 1 hour, images of the tissue samples were collected. A Leica SP8 laser-scanning confocal microscope was used to collect the images, with settings that have been described previously (Welker and Levy, 2022). Three images were taken from the central region of each tissue sample. Using a FIJI macro, counts of callose deposits were obtained as described previously (Welker et al., 2022).

### Statistical analysis

The data were analyzed using JMP Pro v16 software, with a *post hoc* Tukey–Kramer honestly significant difference (HSD) test or t tests to compare the means of the different treatments. Statistical significance was established at *P* < 0.05. Pearson Correlation Coefficient (r) was calculated to validate the modulation in gene expression for RNAseq data and quantitative PCR using JMP Pro v16 software. As for statistical Testing of Phloem Callose Deposits, ANOVA was performed using R statistical software to assess the significance of the model and interactions, any non-zero counts of callose plugs were analyzed with a negative binomial regression in R (R Core Team, 2013). After log-transforming the counts to meet the assumption of normality, ANOVA was performed on the mean counts of each tissue type group.

## Results

### Finger lime trees have enhanced tolerance to *Ca*Las

**Figure 1:**
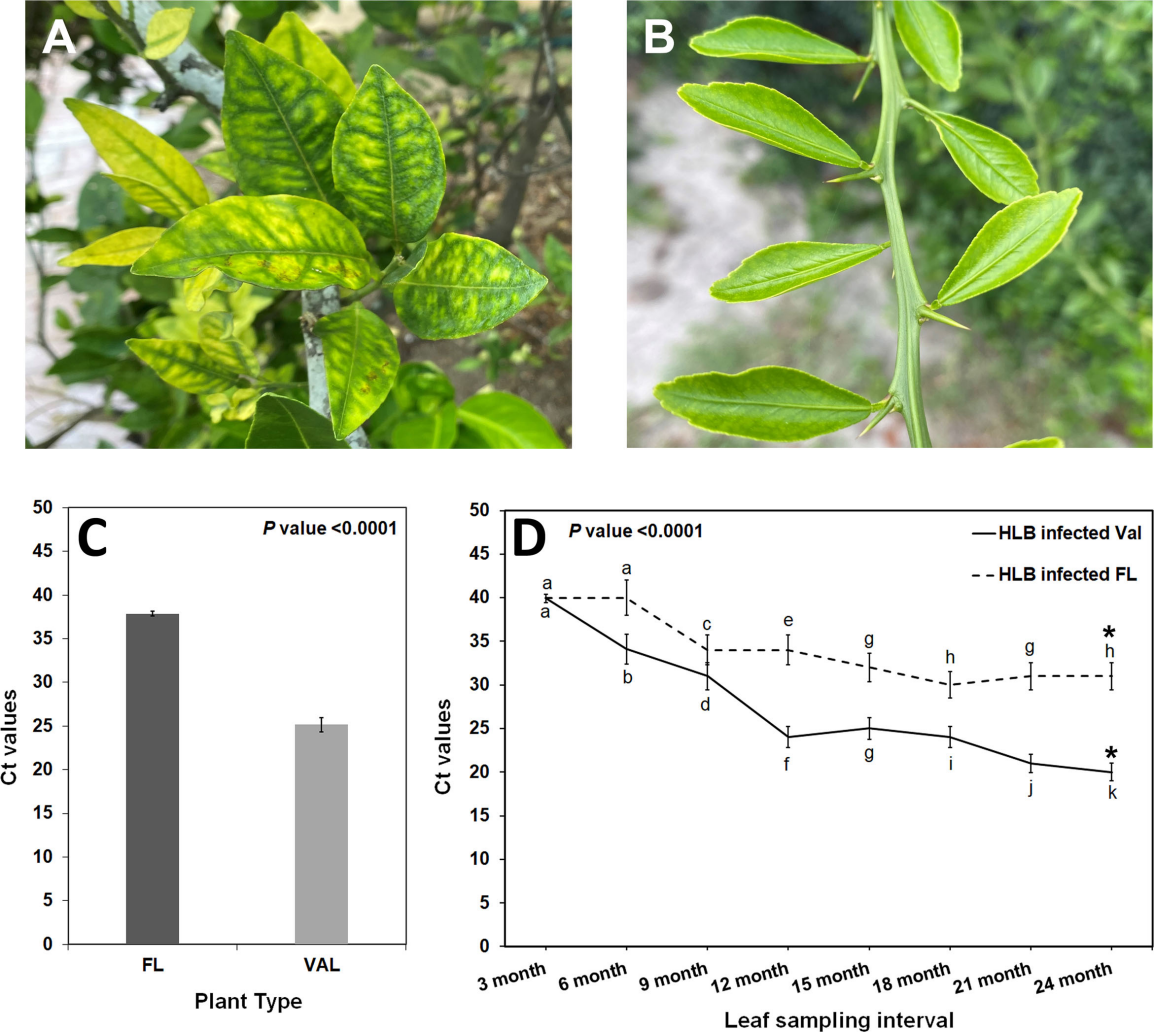
**(A)** HLB infected ‘Valencia’ sweet orange in the field exhibiting the characteristic blotchy mottle pattern in the leaves. **(B)** Finger lime leaves from trees growing in the field with no visible disease symptom. **(C)** Detection of CaLas in leaf tissues of Finger lime and ‘Valencia’ trees by qPCR. Leaf samples were collected from 8-year-old field trees at the beginning of the study. **(D)** CaLas detection from leaf samples collected periodically from trees, side grafted with HLB infected budwood and growing in the green house. * represents the sampling time for RNAseq analysis. Different letters above the error bar indicate statistically significant differences, while the same letters signify no significant differences using the Tukey–Kramer honestly significant difference test (Tukey HSD; p <0.05).

To understand HLB levels in mature finger lime and ‘Valencia’ sweet orange trees growing in the field, leaf samples were collected from 8-year-mature trees, and the total DNA obtained from leaf petioles and midribs was analyzed using qPCR. Our results indicate that the field-grown finger lime trees were always HLB negative (undetermined cycle threshold (Ct) or had high Ct values (37.88 ± 0.28)), whereas ‘Valencia’ sweet orange trees had low Ct values of 25.14 ± 0.82 (**Figure 1C**), indicating active HLB infection.

Subsequently, field-collected HLB-infected ‘Valencia’ sweet orange scions were side grafted onto healthy one-year-mature grafted finger lime and ‘Valencia’ sweet orange trees to observe disease progression under controlled conditions. To assess bacterial population levels in the leaves, qPCR was periodically performed to screen the *Ca*Las titer in the samples collected from greenhouse trees **Table S1**. Screening for the presence of *Ca*Las revealed that when trees were forcibly inoculated with *Ca*Las, both finger lime and ‘Valencia’ sweet orange trees were infected (**Figure 1D**). However, the rate of infection differed between the two accessions. At 24 months following infection, the Ct value of the finger lime trees was, on average, 33.2 ± 1.3, while the ‘Valencia’ sweet orange Ct value was, on average, 23.4 ± 2.8.

**Figure 2:**
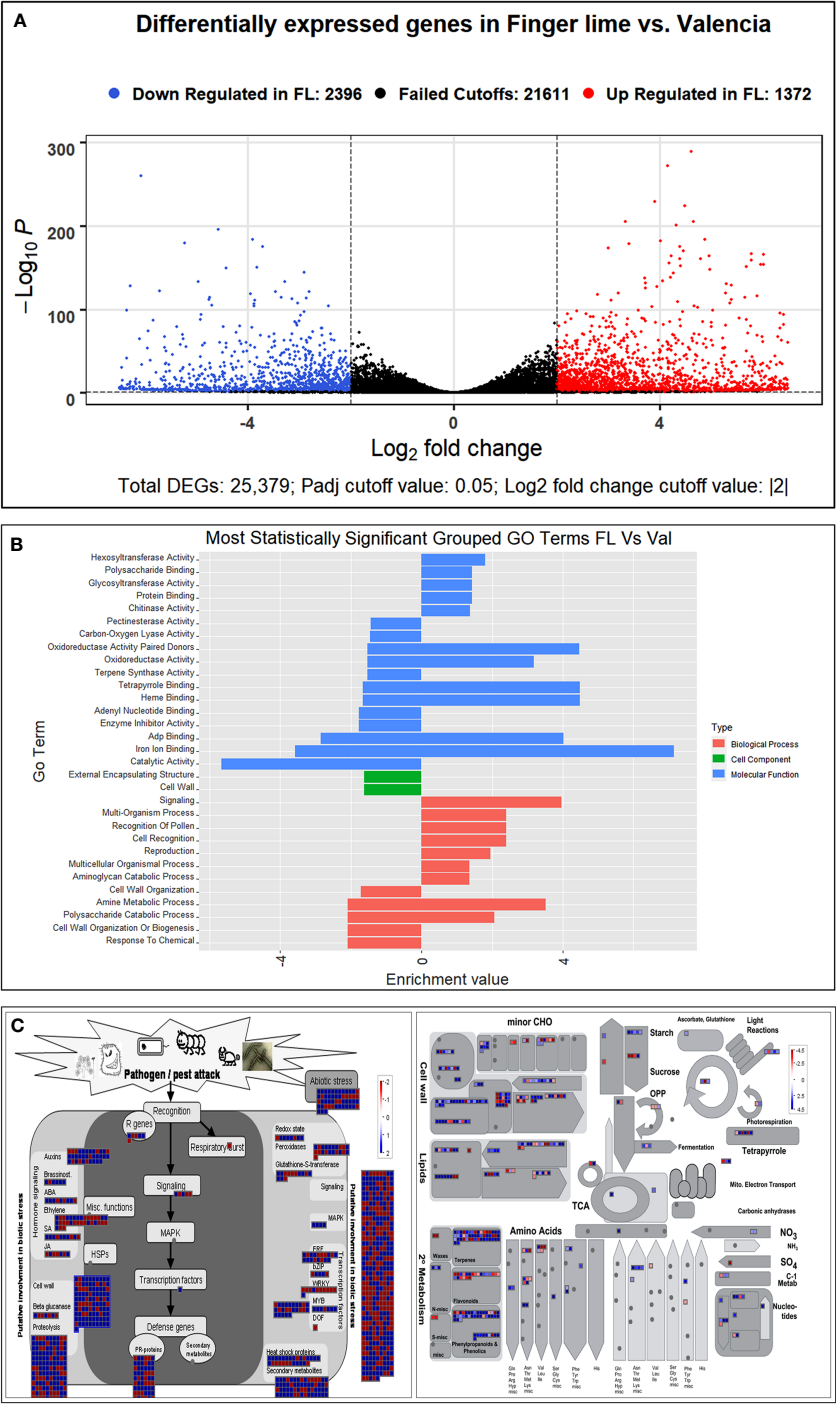
**(A)** Volcano plot of the upregulated and downregulated DEGs. Genes with an adjusted p value of less than 0.05 found with DESeq were assigned as differentially expressed. **(B)** Graphical view of the most statistically significant upregulated and downregulated enriched GO terms in Finger line trees as compared to ‘Valencia’ trees. Statistically significant DEGs were analyzed using AgriGO v2 and REVIGO. **(C)** Differentially expressed genes as identified following MAPMAN analysis. Regulation of stress-related gene pathways by *Ca*Las infection in the infected Finger Lime (Left). Overview of the differentially expressed genes related to the metabolic pathways in Finger lime and ‘Valencia’ sweet orange (Right). Genes that were significantly upregulated following *Ca*Las infection are displayed in blue, and downregulated genes are displayed in red.

A transcriptome analysis was subsequently conducted to understand the possible biological reasons for the HLB tolerance of finger lime compared with susceptible citrus such as ‘Valencia’ sweet orange. The RNA from *Ca*Las-infected samples of finger lime and ‘Valencia’ sweet orange (three technical replicates each) was sequenced using the Illumina HiSeq next-generation sequencing platform. The average total number of raw reads produced was 37,550,067 and 31,506,847 for the *Ca*Las-infected finger lime replicates and the *Ca*Las-infected ‘Valencia’ sweet orange replicates, respectively (**Table 1**); the cleaning process of the reads resulted in average numbers of reads of 29,854,551 (79.51%) and 26,230,524 (83.25%), respectively. The cleaned reads were mapped onto the *C. sinensis* genome. Genomic mapping of the RNA reads revealed that, on average, 24,807,563 (84.84%) and 22,595,692 (86.14%) clean reads were mapped. The mapped reads were analyzed by differential expression analysis. After filtering the differentially expressed genes (DEGs) according to |log_2_(fold-change)| < 2 and an adjusted *P* value (FDR) ≥ 0.05, 3,768 remained. Of the 3,768 DEGs, 2,396 were downregulated in HLB-infected finger lime compared to HLB-infected ‘Valencia’ sweet orange, while 1,372 were upregulated (**Figure 2A**).

**Table 1:** Summary of sequencing, cleaning, and mapping of reads following sequencing the HLB infected finger lime and HLB infected ‘Valencia’ samples.

Run Name	Raw read count	Read count after cleaning	Surviving Read Percent	Mapped reads	Mapped reads percent
FL1	42,957,054	31,809,635	74.05%	26,440,485	83.12%
FL2	34,840,983	28,780,882	82.61%	23,935,454	83.16%
FL3	34,852,164	28,973,136	83.13%	24,046,751	83.00%
FL Avg	37,550,067	29,854,551	79.51%	24,807,563	83.09%
Val1	32,285,138	26,821,818	83.08%	22,756,095	84.84%
Val2	32,676,339	27,320,775	83.61%	23,775,386	87.02%
Val3	29,559,065	24,548,978	83.05%	21,255,596	86.58%
Val Avg	31,506,847	26,230,524	83.25%	22,595,692	86.14%

### Domain differences between finger lime and ‘valencia’ sweet orange infected with HLB

There were 20 GO categories that were significantly upregulated, and 19 GO categories downregulated between the HLB-infected finger lime samples and the HLB-infected ‘Valencia’ sweet orange samples (**Table 2**). Among the domains of the upregulated DEGs, eleven were representatives of the molecular function category. Of these 11 domains, the most significant were heme binding (GO:0020037), tetrapyrrole binding (GO:0046906), and iron ion binding (GO:0005506). The other domains of the upregulated DEGs represented types of biological processes: signaling (GO:0023052), amine metabolic process (GO:0009308), and cell recognition (GO:0008037). Other domains with lower enrichment values are listed in **Table 2**. There were no domains of upregulated DEGs within the cell component category.

**Table 2:** Significant GO terms represented in the HLB infected finger lime *vs* HLB infected ‘Valencia’ comparison.

GoTerm	Type	Description	PosCount	PosEV	NegCount	NegEV
GO:0003824	F	catalytic activity	0	0	655	-5.6576
GO:0005506	F	iron ion binding	57	-7.1549	63	-3.585
GO:0043531	F	ADP binding	56	-4.0269	72	-2.8539
GO:0009308	P	amine metabolic process	13	-3.5229	13	-2.0915
GO:0000272	P	polysaccharide catabolic process	6	-2.0655	8	-2.0915
GO:0042221	P	response to chemical	0	0	33	-2.0915
GO:0071554	P	cell wall organization or biogenesis	0	0	24	-2.0915
GO:0004857	F	enzyme inhibitor activity	0	0	25	-1.7696
GO:0030554	F	adenyl nucleotide binding	0	0	205	-1.7696
GO:0071555	P	cell wall organization	0	0	18	-1.7212
GO:0020037	F	heme binding	54	-4.4815	60	-1.6576
GO:0046906	F	tetrapyrrole binding	54	-4.4815	60	-1.6576
GO:0005618	C	cell wall	0	0	215	-1.6198
GO:0030312	C	external encapsulating structure	0	0	19	-1.6198
GO:0016705	F	oxidoreductase activity paired donors	48	-4.4685	52	-1.5376
GO:0016491	F	oxidoreductase activity	48	-3.1739	153	-1.5376
GO:0010333	F	terpene synthase activity	0	0	17	-1.5376
GO:0016838	F	carbon-oxygen lyase activity	0	0	17	-1.4559
GO:0030599	F	pectinesterase activity	0	0	15	-1.4437
GO:0023052	P	Signaling	44	-3.9586	0	0
GO:0008037	P	cell recognition	16	-2.3979	0	0
GO:0048544	P	recognition of pollen	16	-2.3979	0	0
GO:0051704	P	multi-organism process	17	-2.3979	0	0
GO:0000003	P	Reproduction	16	-1.9586	0	0
GO:0016758	F	hexosyltransferase activity	41	-1.7959	0	0
GO:0005515	F	protein binding	202	-1.4318	0	0
GO:0016757	F	glycosyltransferase activity	44	-1.4318	0	0
GO:0030247	F	polysaccharide binding	12	-1.4318	0	0
GO:0004568	F	chitinase activity	6	-1.3768	0	0
GO:0006026	P	aminoglycan catabolic process	6	-1.3665	0	0
GO:0032501	P	multicellular organismal process	17	-1.3665	0	0

The top downregulated GO terms of the molecular function category included “catalytic activity” (GO: 0003824) and “ADP binding” (GO: 0043531). “Amine metabolic process” (GO:0009308), “polysaccharide catabolic process” (GO:0000272), and “response to chemical” (GO:0042221) were the top downregulated GO terms in the biological process category. Unlike the upregulated GO terms, the downregulated GO terms were assigned to two cellular component categories: “cell wall” (GO: 0005618) and “external encapsulating structure” (GO: 0030312). A graphical view of these data can be found in **Figure 2B**.

### Functional differences between HLB-infected finger lime and ‘valencia’ sweet orange

DEG functional analysis is important to understand the biochemical responses elicited by these genes and the roles they play in the overall function of the plant. The significant DEGs were analyzed *via* PageMan to investigate the functional categories. Out of the 3,768 DEGs, 1,162 were assigned to disease response categories. Nearly all the DEGs belonged to one of the following categories: cell wall, β-glucanase, proteolysis, R genes, signaling, respiratory burst, abiotic stress, redox state, peroxidases, glutathione-S-transferase, secondary metabolites, and pathogenesis-related (PR) proteins **Figure 2C**. When comparing the DEGs in functional categories between HLB-infected finger lime and HLB-infected ‘Valencia’ sweet orange, we found that that genes involved in flavonoids, isoflavones, cytokinin synthesis and degradation, ethylene synthesis and degradation, sugar and nutrient signaling, and the transport of sugars as well as genes encoding isoflavone reductase, UDP glucosyl and glucoronyl transferases, cytochrome p450, GRAS transcription factors, MAD box transcription factors, WRKY transcription factors, DNA methyltransferase, ubiquitin E3, receptor kinases, LRR XI, and DUF were upregulated in finger lime. However, genes assigned to categories related to the cell wall, pectin esterase, pectin methylesterification (PME), phenylpropanoids, lignin biosynthesis, short chain dehydrogenases/reductases, and posttranslational modification of kinases and receptors (such as cytoplasmic kinase VII), as well as a few unassigned ones, were found to be underrepresented in finger lime. Given the functional differences between the upregulated genes among the groups, adding context to better understand relationships within a pathway can help determine the differences between HLB-infected finger lime and HLB-infected ‘Valencia’ sweet orange.

### Pathogen interaction factors were upregulated in finger limes

It has been widely reported that microbe infection induces plant defense through two mechanisms, namely, PTI and ETI, play a substantial role in plant disease resistance (Deng et al., 2018). Seven cell wall LRR family protein-related DEGs were identified as being enriched. Of the DEGs mapped to the genome, 8.0% were kinase-related DEGs. We identified multiple cysteine (Cys)-rich receptor-like protein kinases (CRKs) upregulated in infected finger lime (**Table 3**). No changes in expression (log_2_(fold-change)) were recorded for the mitogen-activated protein kinase-encoding DEG (MAPK) in finger lime and ‘Valencia’ sweet orange when the uninfected tissues were compared with the infected tissues.

**Table 3:** DEGs involved in cysteine-rich receptor-like protein kinase of *Ca*Las infected finger lime and ‘Valencia’ sweet orange.

Gene symbol	Log2 fold change		*Citrus sinensis* ID	*Arabidopsis *homolog
	FL (HLB+)	Val(HLB+)		
Cysteine-rich RLK- 8*	1.55	0.23	orange1.1g007239m	AT4G23160.1
Cysteine-rich RLK- 10	4.17	0.26	orange1.1g041433m	AT4G23180.1
Cysteine-rich RLK- 10	31.5	2.13	orange1.1g041917m	AT4G23180.1
Cysteine-rich RLK- 10	1.45	0	orange1.1g039168m	AT4G23180.1
Cysteine-rich RLK- 10	0.9	9.5	orange1.1g010329m	AT4G23180.1
Cysteine-rich RLK- 10	9.25	0.88	orange1.1g037707m	AT4G23180.1
Cysteine-rich RLK- 10	11.66	0.06	orange1.1g042892m	AT4G23180.1
Cysteine-rich RLK- 10	0.66	0	orange1.1g005893m	AT4G23180.1
Cysteine-rich RLK- 10	14.58	3.17	orange1.1g009186m	AT4G23180.1
Cysteine-rich RLK- 16	5.5	0.69	orange1.1g021682m	AT4G23130.2
Cysteine-rich RLK- 18	2.25	0.09	orange1.1g040682m	AT4G23260.1
Cysteine-rich RLK- 25	2.03	0.02	orange1.1g017211m	AT4G05200.1
Cysteine-rich RLK- 25	5.06	0.6	orange1.1g017150m	AT4G05200.1
Cysteine-rich RLK- 25	2.03	0.02	orange1.1g017211m	AT4G05200.1
Cysteine-rich RLK- 34	7.49	1.47	orange1.1g006125m	AT4G11530.1

*Cysteine-rich RLK: cysteine-rich receptor-like protein kinase.

**Figure 3:**
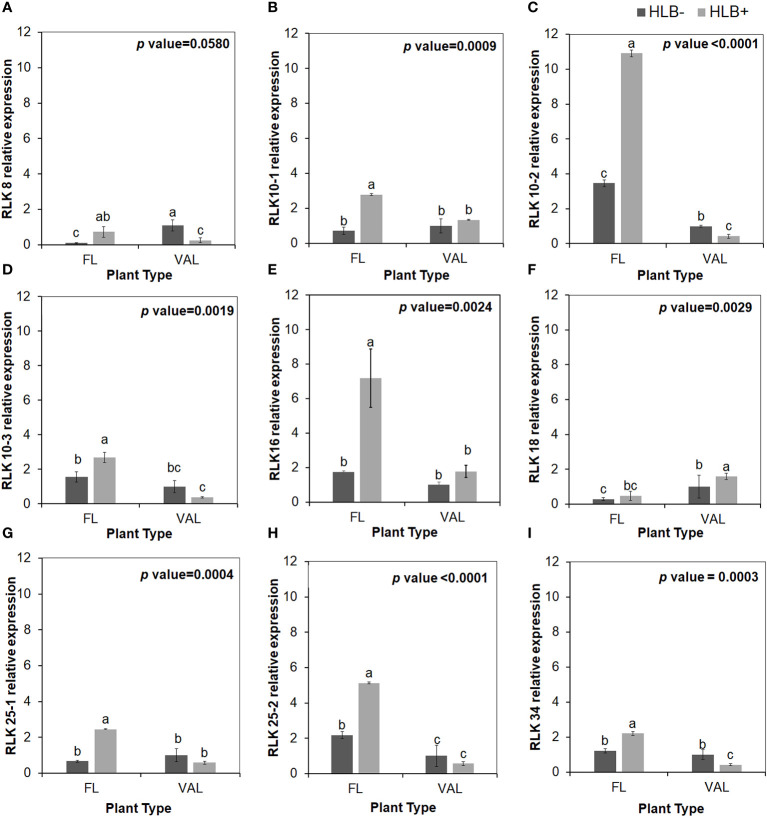
Relative transcript levels of cysteine-rich RLK (RECEPTOR-like protein kinase) as calculated by real-time PCR compared with the CaLas free ‘Valencia’. The CaLas infected samples were collected from five year old trees growing in the field and the CaLas free (control) samples were collected from trees growing in a protected greenhouse. **(A–I)** Relative RLK expression as detected in this study compared with CaLas free 'Valencia'. The control trees were confirmed negative for CaLas before further comparison. Data are means ± SE of twelve samples. Different letters above the error bar indicate statistically significant differences, while the same letters signify no significant differences using the Tukey–Kramer honestly significant difference test (Tukey HSD; p <0.05).

To validate the reliability of the RNA-seq data in terms of the overexpression of CRKs, we randomly selected 9 DEGs encoding Cys-rich RLKs for confirmation by qPCR (**Figure 3**) on samples of infected finger lime and ‘Valencia’ sweet orange obtained from the field. The response of the infected trees in the field was largely consistent with data obtained from the greenhouse trees, indicating that the RNA-seq data reported here are consistent for both sets of samples (greenhouse and field samples).

Several DEGs encoding many other R proteins that predominantly contain a nucleotide-binding site (NBS) and/or LRR domain were differentially expressed in *Ca*Las-infected finger lime. Indeed, 114 pathogenesis-associated DEGs constituted 3% of all the DEGs mapped, 48 of which were upregulated. Twenty-nine of these upregulated genes were PR protein-encoding DEGs of the Tir-NBS-LRR class, 4 were of the NBS-LRR class, and 7 were of the CC-NBS-LRR class. In addition, there were 33 Tir-NBS-LRR class genes, 7 CC-NBS-LRR gene class genes, and 14 NBS-LRR class genes that were downregulated. Transcript levels of orange1.1g035344m, similar to the stable antimicrobial peptide (SAMP) (Huang et al., 2021b) was not detected in our RNAseq DEG data although orange1.1g033887m, an alternate isoform was upregulated in HLB+ ‘Valencia’.

**Figure 4:**
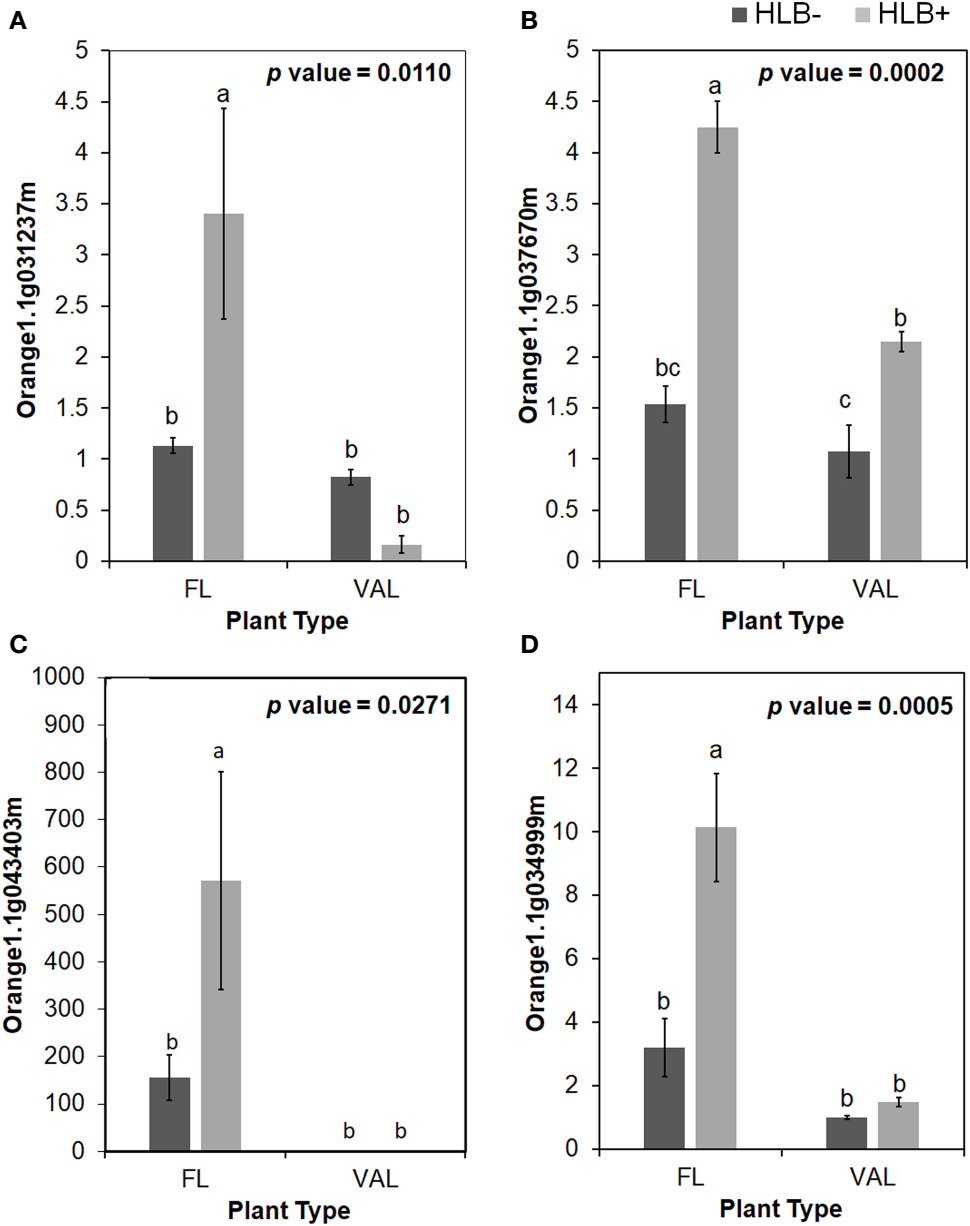
Relative transcript levels of CAP (Cysteine-rich secretory proteins; Antigen 5; and Pathogenesis-related 1 protein) superfamily protein is calculated by real-time PCR compared with the *Ca*Las free ‘Valencia’. **(A)** CAP1, **(B)** CAP2, **(C)** CAP3 and **(D)** LCR69. The *Ca*Las infected samples were collected from five years old samples growing in the field and the *Ca*Las free (control) samples were collected from a protected greenhouse. The control samples were confirmed negative for *Ca*Las before further comparison. Data are means ± SE of twelve samples. Different letters above the error bar indicate statistically significant differences, while the same letters signify no significant differences using the Tukey-Kramer honestly significant difference test (Tukey HSD; p <0.05).

Among these proteins, PR proteins and Cys-rich secretory proteins are thought to be involved in the plant defense response to pathogen infection and plant tolerance (Van Loon, 1997; Baindara et al., 2017). Interestingly, we identified candidate genes encoding several Cys-rich secretory proteins or Pathogenesis-related 1 protein (PR1-like) that were highly upregulated in the infected finger lime relative to *Ca*Las-uninfected and infected ‘Valencia’ sweet orange (**Table 4**). Of these Cys-rich secretory proteins, orange1.1g043403m was highly upregulated in the non-infected finger lime trees, and the expression was recorded as two-fold increase after *Ca*Las infection. The results of the field experiment confirmed this gene was highly upregulated in finger lime trees when compared with the infected ‘Valencia’ sweet orange (**Figure 4**). Additionally, we investigated the presence of this gene (orange1.1g043403m) in several field grown citrus species and cultivars, and we detected lower relative expression in all the evaluated trees compared to finger lime. The only species that presented high expression was the Australian desert lime (*Citrus glauca*), and this expression was negatively associated with *Ca*Las presence (**Figures S3, S4**). In contrast, multiple citrus relatives, such as kumquat (Nagami (*Fortunella margarita*) and Meiwa (*Fortunella crassifolia*)), *Poncirus trifoliata* (50-7 and Flying Dragon), Mandarin (Ponkan and Cleopatra (*Citrus reticulata*), lime (key lime (*Citrus aurantifolia*) and Rangpur lime (*Citrus limonia*)), pummelo (Hirado Buntan pummelo and Siamese Sweet pummelo (*Citrus maxima*)), grapefruit (Ruby Red and Duncan (*Citrus* × *paradisi*)), sweet orange (‘Valencia’ and Parson Brown (*Citrus sinensis*)), Volkamer lemon (*Citrus volkameriana*), Sydney hybrid (*Citrus* x *virgata*), *Citrus papuana*, and *Citrus inodora*, showed lower expression of this gene (orange1.1g043403m) than did finger lime (**Figure S3**). Although *Poncirus trifoliata* is highly tolerant to HLB (**Figure S4**), the relative expression of the Cys-rich secretory protein transcripts identified in this study was lower than that recorded in finger lime.

**Table 4:** DEGs involved in Cysteine-rich secretory proteins or Pathogenesis-related protein of *Ca*Las in finger lime and ‘Valencia’ sweet orange.

Gene symbol	Log2 fold change		Gene function	*Citrus sinensis* ID	*Arabidopsis *homolog
	FL (HLB+)	Val (HLB+)			
*CAP1*	1.83	0.25	**Pathogenesis-related proteins**	orange1.1g031237m	AT4G33720.1
*CAP2*	5330	15.35		orange1.1g043403m	AT4G33720.1
*CAP3*	0.22	15.55		orange1.1g037670m	AT5G66590.1
*LCR69*	41.64	6.89		orange1.1g034999m	AT2G02100.1

CAP, Cysteine-rich secretory proteins; Antigen 5; and Pathogenesis-related 1 protein) superfamily protein, LCR69: low-molecular-weight cysteine-rich 69.

Additionally, many of the DEGs characterized encoded transcription factors, kinase activity-related proteins, or were involved in proteolysis. Sixty-seven DEGs were categorized as encoding transcription factors involved in the biotic stress response, constituting 1.8% of all the mapped DEGs, 27 of which were found to be upregulated in finger lime. The most notable were MYB, WRKY, zinc-finger, and bZIP transcription factors. There were 32 DEGs related to the Myb domain transcription factor family, which are involved in secondary metabolism, hormone signal transduction, plant development, abiotic stress tolerance, and disease resistance, six of which were upregulated (MYB12, MYB17, MYB62, MYB38, MYB102, and MYB112). Eleven of the DEGs identified belonged to the WRKY transcription factor family, the members of which play important roles in plant development and stress responses. Nine of the WRKY transcription factors were upregulated (WRKY14, WRKY23, WRKY28, WRKY31, WRKY47, WRKY48, WRKY50, WRKY72 and WRKY75). Additionally, a Dof-Type zinc finger DNA-binding family protein (DAG1) and two BZIP transcription factor family proteins (TGA9 and BZIP42) were found to be upregulated in finger lime, while bZIP58, BZIP61, and PAN were downregulated.

### Genes involved in hormone signaling pathways were differentially expressed

Hormone signaling is important in plant physiology and regulates many aspects from the transduction of messages through plants to adaptation to the environment to the timing of fruit development and ripening. Out of the mapped genes, 2.6% (99 DEGs) were associated with hormone synthesis. Nine abscisic acid-induced genes were identified. Three of these were Hva22-like protein-encoding DEGs (orange1.1g042117m, orange1.1g038094m, and orange1.1g030361m) and were upregulated. Two Gram Domain-Containing Protein 2-encoded DEGs were downregulated. Abscisic acid degradation-related DEGs were downregulated only in finger lime; these genes included epoxycarotenoid dioxygenase, aldehyde oxidase, and zeaxanthin oxidase. Auxin induced-regulated related DEGs were expressed to a higher degree in ‘Valencia’ sweet orange than in finger lime, with 25 out of the 34 DEGs upregulated in ‘Valencia’ sweet orange. The upregulated genes in finger lime included those encoding ILR1, TIR1, DFL1, and GH3.1 proteins. Of the six brassinosteroid-related DEGs, cytochrome P450 (orange1.1g037705m) was upregulated, while the STE1-, HYD1-, and SMT1-encoding genes were downregulated.

There were 33 ethylene-related DEGs mapped, 17 of which were upregulated, including Gibberellin 2-Oxidase, 2-Oxoglutarate, Kar-Up Oxidoreductase 1, Integrase-Type DNA-Binding, GASA1 and SRG1. Seven jasmonate-related genes were also identified, which were upregulated and included three of LOX2 and JAZ1 gene. Finally, we identified ten salicylic acid (SA)-related genes, and three genes encoding methyltransferase were found to be upregulated, namely, orange1.1g017363m, orange1.1g044676m, and orange1.1g043411m in the infected finger lime.

### Protease related genes were generally downregulated in the finger lime

Among the DEGs mapped, 139 were associated with biotic proteolysis, accounting for 3.6% of all the mapped genes. Fifty-one of these genes, including orange1.1g044297m, which encodes a P-Loop containing Nucleoside Triphosphate Hydrolases Superfamily Protein, were upregulated in finger lime. However, the Aaa-type gene Aaa-Atpase 1, several Cytochrome Bc1 synthesis-related genes, aspartate protease, autophagy-related DEGs, and a metalloprotease-related DEG were downregulated. Of the 10 Cys protease-related DEGs that were mapped, only one of the Cys protease inhibitor-encoding genes (orange1.1g018968m) was upregulated in finger lime. There were 11 serine protease (SP)-encoding, 2 Kunitz Family Trypsin- and protease inhibitor protein-encoding, and 15 Subtilase-related DEGS downregulated in finger lime. In contrast, 20 Ubiquitin E3 Scf F-box and one Ubiquitin E3 Scf Skp (orange1.1g030652m) genes were overexpressed in finger lime, while 16 Ubiquitin E3 Ring, 1 Ubiquitin E2, 2 Ubiquitin Proteasome, 1 Ubiquitin 4, and 1 Polyubiquitin 10 genes were downregulated.

We detected none to minimal expression of three xylem Cys protease 1 (XCP1) and one of xylem Cys protease 2 encoding genes in the finger lime. Comparatively, these genes were upregulated in response to *Ca*Las infection in ‘Valencia’ sweet orange. In contrast, one Xylem Serine Peptidase 1 gene (orange1.1g004503m) was highly upregulated in the infected finger lime trees (**Table 5**).

**Table 5:** DEGs involved in cysteine protease production in finger lime and ‘Valencia’ sweet orange.

Gene symbol*	Log2 fold change		Gene function	*Citrus sinensis ID*	*Arabidopsis* homolog
	FL (HLB+)	Val (HLB+)			
XCP1	0	5.02	cysteine proteases	orange1.1g048025m	AT4G35350.1
XCP1	0.07	1.83		orange1.1g018781m	AT4G35350.1
XCP1	0.08	17.44		orange1.1g019063m	AT3G49340.1
XCP2	0.47	18.78		orange1.1g018649m	AT1G20850.1
CP	0.03	12.78		orange1.1g032006m	AT4G15880.1
CP	2.12	34.03		orange1.1g028661m	AT1G50670.1
CP	0.05	16.56		orange1.1g019112m	AT3G49340.1
XSP 1	98.82	10.01	xylem serine peptidase 1	orange1.1g004503m	AT4G00230.1

*XCP1- xylem cysteine Protease 1, XCP2- xylem cysteine Protease 2 and CP- Cys protease.

**Figure 5:**
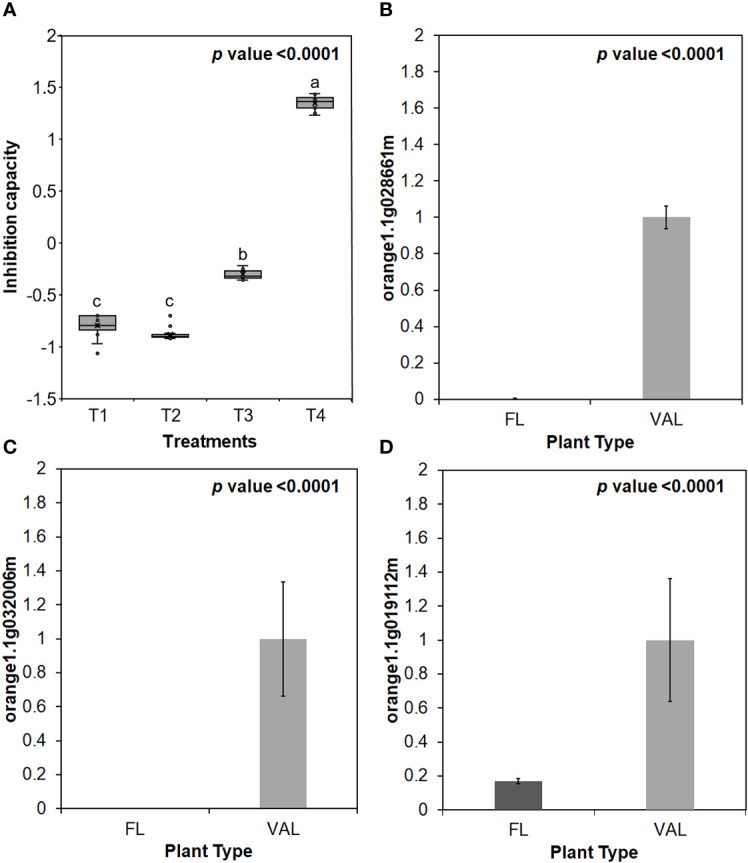
**(A)** Inhibition capacity of Finger lime and ‘Valencia’ T1- FL (HLB+) + substrate, T2- FL (HLB+) + substrate, T3- VAL (HLB+) + substrate + E64 and T4- Val (HLB+) + substrate + E64. The inhibition capacity was compared with the HLB negative leaves. Relative transcript levels of Cysteine proteinases superfamily protein transcription factors were calculated by real-time PCR and compared with the *Ca*Las free ‘Valencia’. The *Ca*Las infected trees were collected from five year old trees growing in the field and the *Ca*Las free (control) samples here instead of trees were collected from trees kept in a protected greenhouse. The control trees were confirmed negative for *Ca*Las before further comparison. Data are means ±SE of twelve samples. **(B–D)** Relative gene expression of selected cysteine proteases genes in the infected finger lime compared with infected 'Valencia'. Different letters above the error bar indicate statistically significant differences, while the same letters signify no significant differences using the Tukey-Kramer honestly significant difference test (Tukey HSD; p <0.05).

Additionally, the infected ‘Valencia’ sweet orange trees showed higher inhibition capacity of E64 compared with that of the finger lime trees and *Ca*Las-free trees (**Figure 5A**). We selected some of Cys protease transcription factors for validation *via* real-time PCR. We found that the relative expression of genes encoding three transcription factors of Cys protease was downregulated following *Ca*Las infection in finger lime compared with ‘Valencia’ sweet orange (**Figuress 5B–D**).

### Genes involved in cellular development were differentially regulated

One hundred and one DEGs related to processes involved in cell wall synthesis and support were identified from our transcriptome data. Only 25 of these genes were upregulated in finger lime. Ten cellulose synthase-encoding genes were mapped, of which those encoding D1, G2, and G3 cellulose synthases were found to be upregulated compared to those of ‘Valencia’ sweet orange. Five genes related to cellulase and 1,4-β-glucanase degradation, namely, orange1.1g042201m, orange1.1g036635m, orange1.1g041590m, orange1.1g010632m, and orange1.1g048736m, were upregulated in finger lime. Expansin A1 (orange1.1g025919m) and A20 (orange1.1g025617m) were also upregulated in finger lime. Of the 17 PME-related genes identified by mapping, the PME inhibitor (orange1.1g010441m) was the only DEG upregulated in finger lime. Additionally, six arabinogalactan protein (AGP)-related DEGs were identified, five of which were downregulated, while the gene encoding FLA3 protein (orange1.1g042255m) was upregulated.

### qPCR validated the RNA-seq data

**Figure 6:**
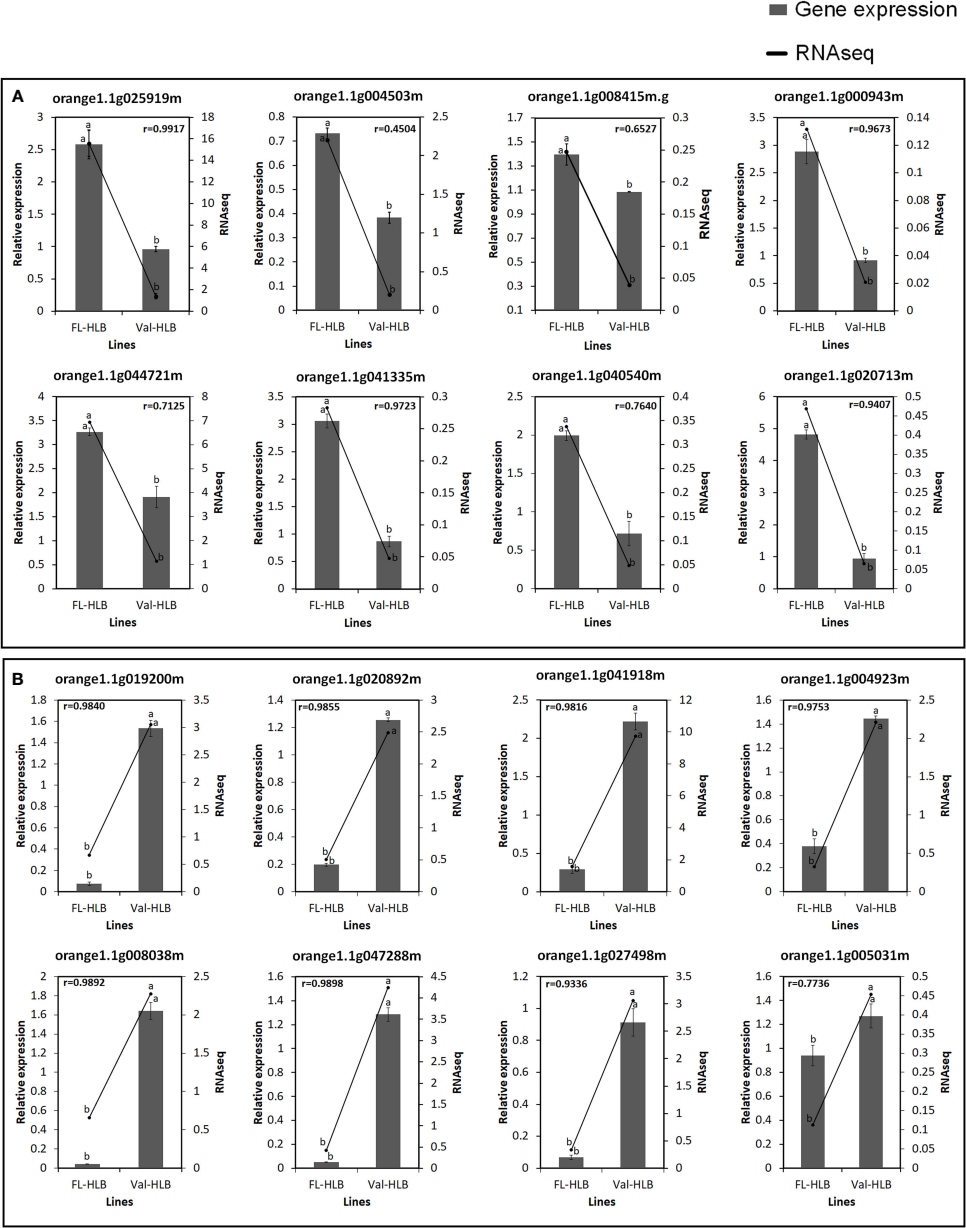
Verification of expression levels of selected upregulated **(A)** or downregulated **(B)** DEGs in the infected Finger lime (FL-HLB) compared to infected ‘Valencia’ (Val-HLB) as determined by qPCR (2-ΔΔCt). Different letters (a, b) represent a significant difference at *p* ≤ 0.05 using Tukey–Kramer honestly significant difference (HSD) and error bars represent SE (n = 3). Pearson Correlation Coefficient (r)> greater than 0.5 is considered positive and strong.

To evaluate the accuracy of the RNA-seq data and the presence of technical artifacts or errors introduced during the RNA-seq library preparations, the expression of several highly conserved genes and genes encoding transcription factors were assayed through qPCR. Eight of the selected genes (orange1.1g025919m, orange1. 1g004503m, orange1.1g025919m, orange1.1g000943m, orange1.1g044721m, orange1.1g041335m, orange1.1g040540m, orange1.1g020713m, orange1.1g008415m) were upregulated in finger lime (**Figure 6**). However, the RNA-seq data revealed that several genes were downregulated (orange1.1g019200m, orange1.1g020892m, orange1.1g041918m, orange1.1g027498m, orange1.1g004923m, orange1.1g005031m, orange1.1g047288m, orange1.1g008038m) (**Figure 6**). The qPCR results were consistent with the RNA-seq data, and the mRNA expression of these genes was either significantly up- or downregulated in the infected finger lime compared with the infected ‘Valencia’ sweet orange.

### Phloem and xylem morphological differences sheds light on finger lime HLB tolerance

**Figure 7:**
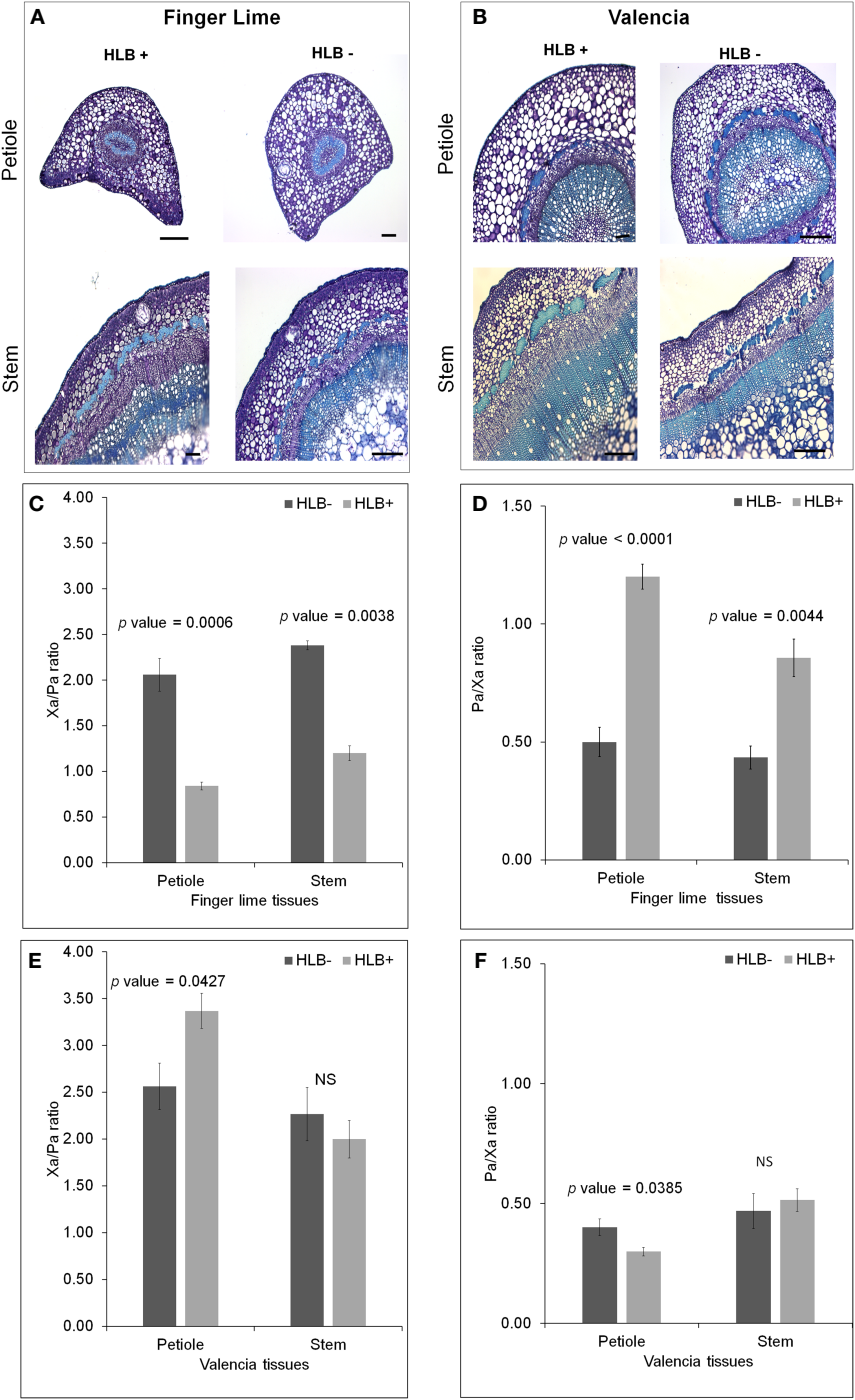
Morphological differences between healthy and HLB-infected Finger lime and ‘Valencia’. Brightfield images of petiole and stems for each healthy and HLB-infected cultivar, Finger lime **(A)**, ‘Valencia’ **(B)**. Phloem Ratio **(C)** and Xylem Ratio **(D)** in Finger lime and Phloem Ratio **(E)** and Xylem Ratio **(F)** in ‘Valencia’. Bars represent standard error. NS, not significant.

We observed phloem and xylem morphological differences in the *Ca*Las– and *Ca*Las+ petioles of finger lime and ‘Valencia’ sweet orange (**Figures 7A, B**). Because phloem and xylem thickness are dependent on tissue type and age, we calculated a relative phloem thickness (RPT) by dividing the average phloem thickness by the average xylem thickness within the same sample. There were significant differences between healthy and HLB-infected finger lime petioles (*p* value = 0.0001) and between healthy and HLB-infected stems (*p* value = 0.0044) (**Figure 7D**). *Ca*Las-infected finger lime had a significantly higher RPT compared to healthy finger lime. This was also true for ‘Valencia’ sweet orange petioles (*p* value =0.0385) (**Figure 7F**). The xylem/phloem ratio (Xa/Pa) showed significant difference in finger lime in both petioles (*p* value = 0.0006) and stems (*p* value = 0.0038) (**Figure 7C**), however, there was slight differences in Xa/Pa in ‘Valencia’ petioles (*p* value = 0.0427) and no significant difference in the stems (**Figure 7E**). Together, our results suggest that phloem may regenerate in finger lime in response to HLB infection.

### Quantification of phloem callose deposits indicated differential callose deposition

**Figure 8:**
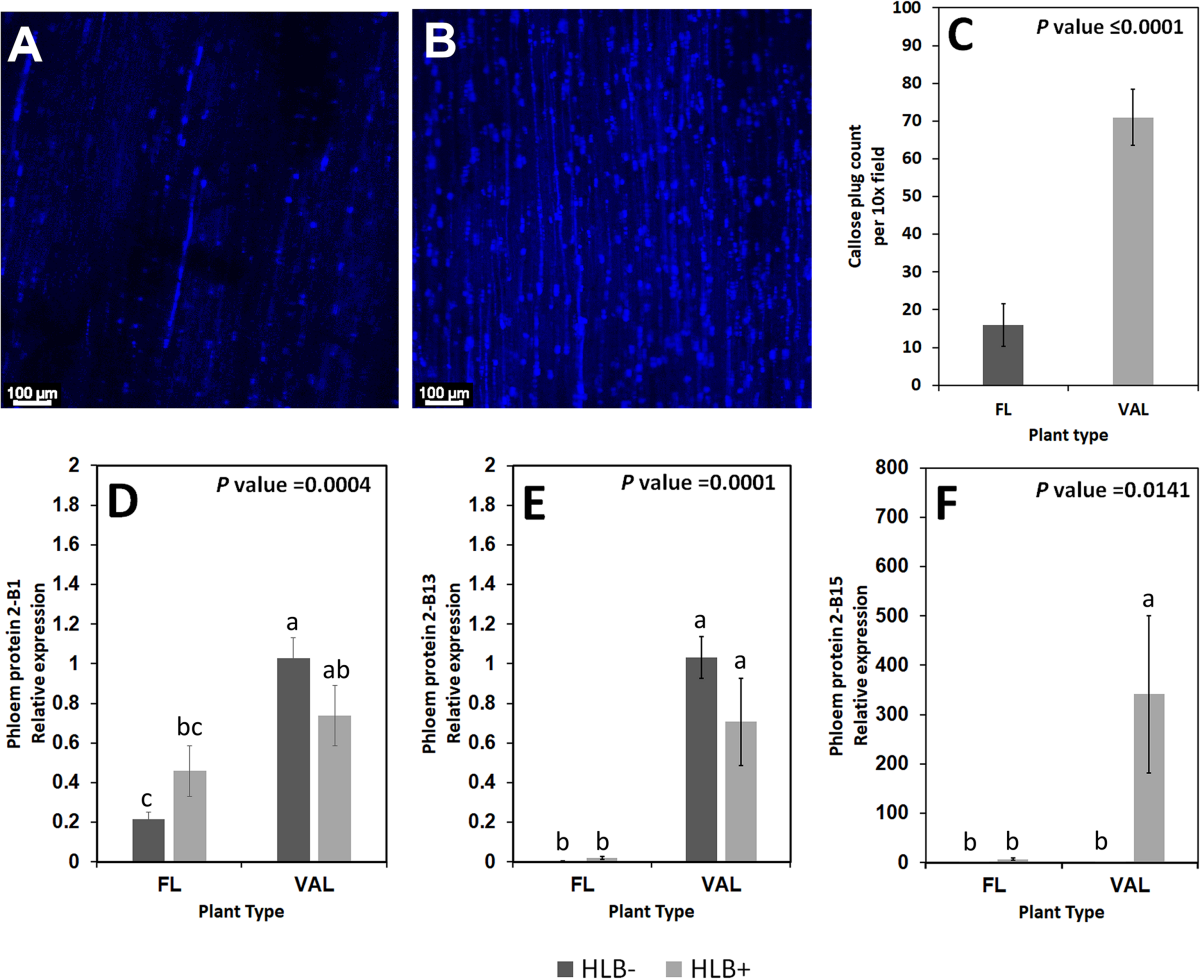
Non-zero counts of callose plugs per 10x field image of the stem phloem of *Ca*Las+ Finger lime **(A)** and ‘Valencia’ sweet orange **(B)** sampled after 2 years following infection. The mean callose formation count per 10x field image was significant. **(C)** Relative transcript levels of phloem proteins are calculated by real-time PCR and compared with the *Ca*Las free ‘Valencia’. The *Ca*Las infected samples were collected from five year old trees growing in the field and the *Ca*Las free (control) samples were collected from trees kept in a protected greenhouse. The control trees were confirmed negative for *Ca*Las before further comparison. Data are means ± SE of twelve samples **(D–F)**. Different letters above the error bar indicate statistically significant differences, while the same letters signify no significant differences using the Tukey-Kramer honestly significant difference test (Tukey HSD; p <0.05).

We found increased accumulation of callose in the infected ‘Valencia’ sweet orange compared with the infected finger lime. Analysis of the callose formation counts revealed a significant difference in the percentage of images with few callose plugs in the stem phloem of *Ca*Las+ ‘Valencia’ sweet orange (0%) and finger lime (26%); **Table 6**. Among the images of phloem that did contain callose formations, ‘Valencia’ sweet orange had significantly higher mean counts of formations per image (**Figures 8A–C**).

**Table 6:** Percent of images with zero callose plugs per 10x field image of the stem phloem of *Ca*Las+ ‘Valencia’ sweet orange and finger lime.

Plant type	Images with zero callose plugs
Finger lime	26%*
Valencia	0%

*p> value <0.001.

### Phloem protein genes are downregulated in finger limes

Some of the transcripts encoding for phloem proteins in the samples were analyzed *via* qPCR. *CsPP2-B1* transcripts were detected in both finger lime and ‘Valencia’ but were not statistically significant when the uninfected and infected trees were compared. *CsPP2-B13* transcripts were almost undetectable in finger lime samples, while they were upregulated in ‘Valencia’. The relative transcript levels of *CsPP2-B15* were highly upregulated in the infected ‘Valencia’ sweet orange leaves compared with the finger lime leaves (**Figures 8D–F**).

## Discussion

HLB is a devastating disease affecting citrus worldwide. Currently, many strategies have been explored for HLB mitigation, including application of antimicrobials, macro, micronutrients and plant defense inducers, control of insect vectors, thermotherapy, biocontrol, and eradication of HLB symptomatic citrus trees (Yang and Ancona, 2022). However, these strategies have shown limited success in field applications, and effective long term HLB management remains a challenge. Thus, breeding for HLB tolerance may provide the most effective and sustainable solution to combat HLB (Bové, 2006). In the field, *Ca*Las-infected ‘Valencia’ sweet orange trees display symptoms of stunted growth, yellow shoots, and blotchy mottled leaves while adjacent finger lime grow normally and produce fruits without any apparent visible HLB symptoms (**Figures 1A, B**). *Diaphorina citri*, the vector transmitting *Ca*Las has a feeding preference for certain citrus cultivars. ‘Valencia’ sweet orange is considered a preferred host while finger limes are considered not suitable (Felisberto et al., 2019). Poor *Diaphorina citri* colonization levels observed on finger lime trees in a recent long term field study confirms these observations (Ramadugu et al., 2016). Under controlled conditions, finger limes trees do get infected following budding with *Ca*Las-infected budwood (Alves et al., 2020). However, the mechanism for this perceived tolerance to *Ca*Las in the host is unknown and our study provides insights into the tolerance mechanism. Finger limes are monoembryonic and open pollinated seedlings can vary in their tolerance to HLB (Ramadugu et al., 2016). Finger lime trees (clone DPI 50-36) have remained HLB negative under field conditions for more than a decade. This study was designed to understand this perceived tolerance by graft inoculating budded trees of the same clone under controlled greenhouse conditions.

The possible genetic mechanisms of the symptoms of *Ca*Las in citrus trees have been previously discussed by several groups (Albrecht and Bowman, 2008; Kim et al., 2009; Rawat et al., 2015; Martinelli and Dandekar, 2017; Curtolo et al., 2020; Ma et al., 2022). Nehela and Killiny (2020) summarized three main potential mechanisms during *Ca*Las infection: (I) disorder of carbohydrate metabolism affecting the flow of nutrients and source–sink disruption due to starch accumulation in leaves; (II) phytohormones alteration in response to stress; and (III) activation of detoxification proteins, particularly glutathione-S-transferases (GSTs) and modulation of antioxidant pathways.

Understanding the virulence mechanisms employed by *Ca*Las against the host is an important step to identify an approach to increase plant defense. Insect-transmitted bacteria such as *Ca*Las utilize the general Sec secretion system to release effectors (Sugio et al., 2011). However, the mechanism of the *Ca*Las effectors secretion remains poorly understood. Protein effectors often suppress plant defense or manipulate developmental processes within the host to benefit the pathogen (Jones and Dangl, 2006). Previous research has shown that *Ca*Las encodes several SDEs, many of which are conserved across *Ca*Las isolates and of which were found to be highly present in infected citrus tissue at a relatively early infection stage (Pagliaccia et al., 2017; Tran et al., 2020).

Several studies have demonstrated that the activity of leucine-rich repeat proteins (LRR-RLKs) serves as an early warning system for detecting the presence of potential pathogens and activating protective immune-related signaling in plants (Matsushima and Miyashita, 2012). Interestingly, receptor kinases, LRRs and cysteine (Cys)-rich receptor-like protein kinases** **(CRKs) were found to be upregulated in HLB infected finger lime. Our findings are similar to those reported by Peng et al. (2020), where the involvement of multiple nucleotide-binding site-containing and LRR-encoding genes in the HLB tolerance/resistance process in *Poncirus trifoliata* was reported. CRKs are characterized by the presence of one to four copies of Domain of Unknown Function 26 (DUF26) and a C–X8–C–X2–C motif in the extracellular receptor region at the N-terminus (Mou et al., 2021). These conserved Cys residues might be required to form the three-dimensional structure of the protein through disulfide bonds (Chen, 2001) and can mediate protein–protein interactions (Rayapuram et al., 2012). Several CRKs have been functionally characterized in response to pathogen infection. We detected overexpression of *CsCRK10, CsCRK16, CsCRK25 and CsCRK34* in the infected finger lime. Functional analysis of the RLKs in *Ca*Las infected plants would be difficult because *Ca*Las is an intracellular bacterium and inoculated directly by the psyllids into the phloem tissues and would not interact with the external receptors of the plant cell. Thus, it has little or no interaction with PTI mechanisms. In *Arabidopsis*, most CRKs are differentially regulated by SA, ROS, and pathogen infection (Czernic et al., 1999; Du and Chen, 2000; Ohtake et al., 2000; Chen et al., 2003; Zhang et al., 2013). Curtolo et al. (2020) reported that the *Ca*Las defense mechanisms were controlled by a class of receptor-related genes and the induction of WRKY transcription factors. These observations suggest that CRKs could play a vital role in the regulatory network regulating the finger lime response to *Ca*Las infection, suggesting that members of the CRK gene family can be effective targets for the improvement of citrus tolerance to HLB disease.

**Figure 9:**
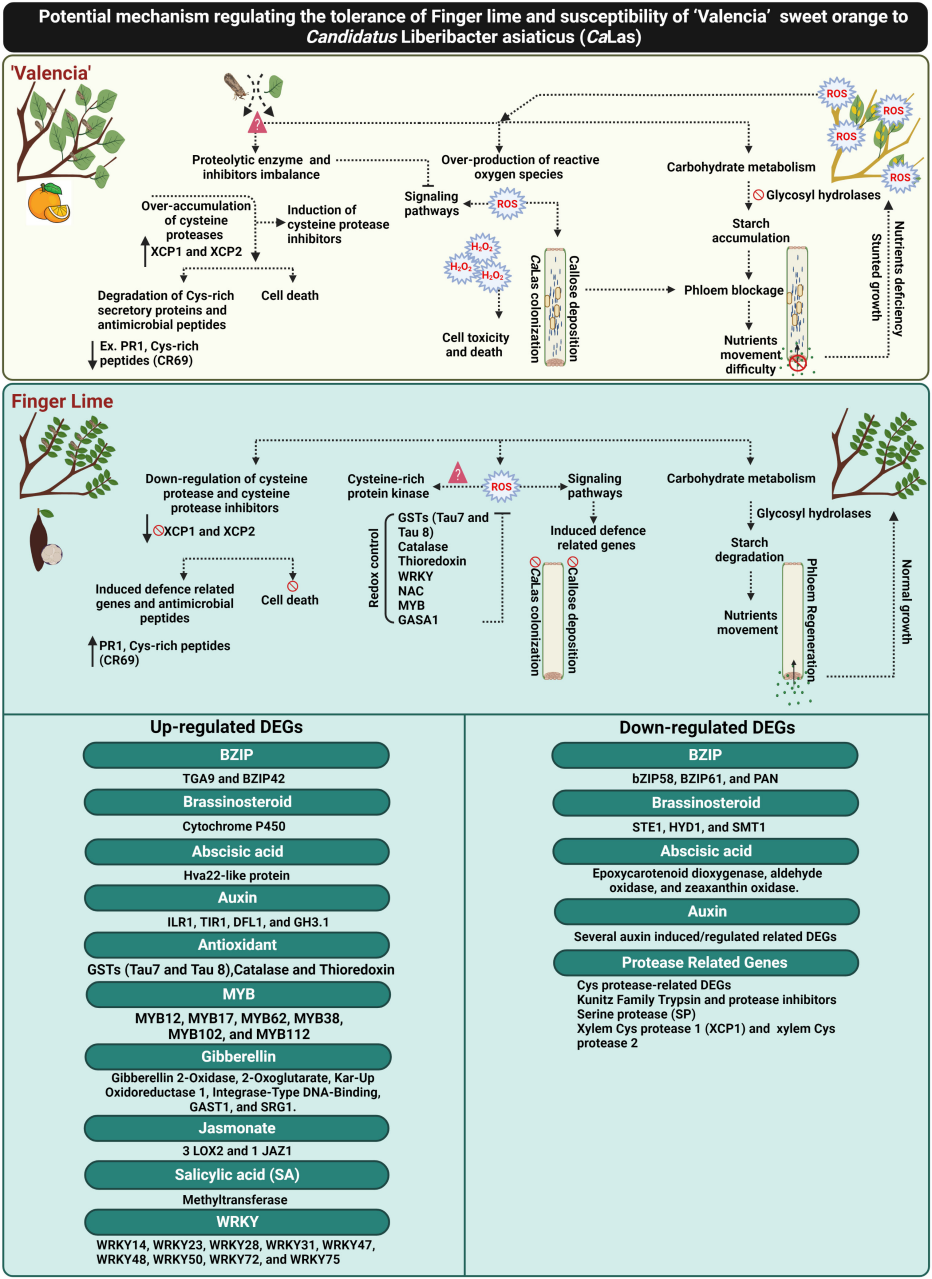
Schematic diagram elucidating the potential mechanism of *Ca*Las tolerance in the Finger lime, and susceptibility in ‘Valencia’ sweet orange. The figure was created in BioRender.com.

Recently, Ma et al. (2022) reported that Huanglongbing (HLB) could by controlled by ROS detoxification *via* induction of antioxidant pathways and plant growth hormones (particularly GA). Additionally, the involvement of GA signaling in HLB resistance has also been reported by Rawat et al. (2015) and Curtolo et al. (2020). Curtolo et al. (2020) suggested that the genetic mechanism of HLB tolerance was associated with the downregulation of gibberellin (GA) synthesis and cell wall strengthening. In the current study, we reported the upregulation of several candidate genes responsible for controlling GA synthesis and production of bioactive GA in the infected finger lime such as gibberellin 2-oxidase 8 (GA2ox8), gibberellin 3-oxidase 1 (GA3ox1) and cysteine-rich protein or GA-stimulated transcript (GAST1 protein homolog 4). Additionally, our data showed that *Ca*Las induced ROS detoxification pathways and activated glutathione-S-transferases (GSTs), catalase and thioredoxin in finger lime. Glutathione-S-transferases was suggested to be as an important modulator of citrus tolerance to HLB disease (Martinelli and Dandekar, 2017). We also recorded the overexpression of several plant growth regulator-related-genes that may have direct or indirect function in ROS mitigation (**Figure 9**).

Plants accumulate inducible defense-related proteins in response to biotic and abiotic stress. Antimicrobial peptides (AMPs) and other Cys-rich peptides are major components of innate immunity in various groups of organisms, including insects, mammals and plants (Pushpanathan et al., 2013; Slavokhotova et al., 2017). Some eukaryotic AMPs are largely Cys-rich peptides, which are defined as defensins (Baindara et al., 2017). A potent AMP has recently been identified from finger lime (Huang et al., 2021b). A Cys-rich secretory protein/PR1-like protein was also found to be abundant in finger lime and in HLB-tolerant Australian desert lime (*C. glauca;* Ramadugu et al. (2016)). We also identified a low-molecular-weight defensin (Cys-rich 69: LCR69) in this study that is highly upregulated in finger lime trees. Defensins usually contain six to eight conserved Cys residues and thus are referred to as Cys-rich peptides (CRPs) (Silverstein et al., 2007).

Cell wall integrity sensing is a mechanism by which plants can be induced to mitigate biotic stress. Various strategies involving resistance of the cell wall against plant pathogens have evolved, such as the remodeling of the cell wall and alterations to cell wall-associated protein distribution and accumulation (Leszczuk et al., 2019). Both cell wall synthesis and proteins associated with the cell wall have roles in structural support, and the presence or absence of these proteins may indicate disease susceptibility or infection. The susceptibility of ‘Valencia’ trees to *Ca*Las infection resulted in a decreased ability to synthesize new cellular components, whereas the finger lime was not affected in the same manner and thus could continue with cell metabolism and growth. These findings are agreed with Fan et al. (2012) who compared resistant rough lemon and susceptible sweet orange following *Ca*Las infection. They reported that cell wall-related pathways were upregulated in rough lemon during the late stage of infection, while there was downregulation in the susceptible sweet orange. Thus, the rough lemon trees generated healthy new growth following infection despite older leaves having some blotchy mottle, while the growth was inhibited in sweet orange trees.

HLB infection induced the differential expression of multiple genes encoding enzymes and proteins involved in the synthesis, assembly, and modification of the cell wall. Overall, many genes involved in cellulose synthesis and 1,4-β-glucan degradation as well as those encoding cellulase glycosyl hydrolases, polygalacturonases and fasciclin-like AGPs were upregulated in the infected finger lime. Several other genes involved in cellulose synthesis, such as cellulose synthase D1, G2 and G3, were also upregulated in *Ca*Las-infected finger lime. *CaLas* infection in ‘Valencia’ sweet orange resulted in the repression of genes encoding cellulose synthase-like D4 (CSLD4) and CSLC7 that mediate synthesis of β-1,4 linkages in the hemicellulose backbones (Aritua et al., 2013). In the phloem, the sieve plate pores accumulate callose, a β-1-3 glucan, and starch, which restrict symplastic transport, in response to *Ca*Las infection (Koh et al., 2012; Welker et al., 2022). Callose deposition and phloem protein (PP) plugging of the sieve tubes are defensive measures to form physical barriers that prevent the spread of pathogens (Musetti et al., 2010). Excessive callose deposition is considered the main reason for phloem blockage in HLB disease. This blockage limits the transfer of organic compounds from the sites of photosynthesis (source) to where photosynthate is stored (sink), which affects plant growth and mimics the symptoms of a nutrient disorder. We observed decreased callose accumulation in finger lime phloem tissue. Previous research revealed that some phloem proteins play an important role in callose deposition (Wen et al., 2018). When the gene expression of HLB infected and healthy controls was compared, PP2 genes were found to be upregulated in the infected trees (Musetti et al., 2010; Ghosh et al., 2018). In the comparison between finger lime and ‘Valencia’ sweet orange, we found that phloem proteins were significantly induced by *Ca*Las infection in ‘Valencia’ sweet orange, while they were repressed in finger lime. Specifically, *CsPP2-B15* was found to be highly upregulated in the infected ‘Valencia’ sweet orange compared to the finger lime. These findings agree with those of Wen et al. (2018), who found that *CsPP2-B15* expression was upregulated in infected leaves of Jincheng orange (*C. sinensis* Osbeck) and downregulated in the HLB tolerant sour pummelo (*C. grandis* Osbeck). Similarly, *CsPP2-15* was also found to be downregulated in the tolerant *C. ichangensis* (Wu et al., 2020). This is in addition to our observations that the ‘Valencia’ sweet orange phloem produces more callose. It should be noted that the enhanced production of callose and phloem proteins in the susceptible plants is purely a mechanical response: it chokes off the transport of nutrients and other vital molecules and at the same time, does not prevent the spread of *Ca*Las. Killiny et al. (2022) suggested that the increased ROS could be one of the factors affecting callose deposition as reported for *Ca*Las-infected citrus trees.

The vascular cambium increases stem diameter *via* periclinal divisions and the circumference by anticlinal divisions, resulting in the development of secondary phloem and xylem (Chaffey, 1999). Proteolytic enzymes, including serine, Cysteine and threonine proteases, have been implicated in the regulation of vascular tissue differentiation. Beers and Zhao (2001) screened seven protease genes, including members of the serine, Cysteine, and aspartic acid protease families, and reported that the expression of three genes (XCP1, XCP2, and XSP1) was xylem specific. XCP1 and XCP2 are predicted to encode papain-like Cys proteases, and XSP1 is predicted to encode a subtilisin-like SP. Clark et al. (2018) evaluated six candidates of Cys protease and reported that the *C. sinensis* protein annotated as “xylem Cys protease 1”, a member of the PLCP family, was confirmed by yeast two-hybrid (Y2H) assays as interacting with *Ca*Las effectors (SDE1). Papain-like Cys proteases are important regulators involved in numerous plant biological processes, including programmed cell death (PCD) and leaf senescence. It has been demonstrated that activation of cysteine proteases induced programmed cell death (PCD) pathway of plant cells (Minami and Fukuda, 1995; Ye and Varner, 1996). Particularly, two xylem-specific PLCPs (*AtXCP1* and *AtXCP2*) were found to be expressed at a high level in xylem during the PCD process (Liu et al., 2018). We did not find any significant expression of many of the xylem Cys protease 1 genes in our finger lime transcriptome data; however, these genes were upregulated in the infected ‘Valencia’ sweet orange. Cys proteases and their inhibitors were highly expressed in ‘Valencia’ sweet orange field samples. Under stress conditions, the activity of PLCPs and their regulatory factors can be disrupted, resulting in unknown consequences at the cellular level (Liu et al., 2018). We suggest that the overexpression of proteases and inhibitors in ‘Valencia’ sweet orange reflects an imbalance in metabolism. *Ca*Las infection can alter the function of the transcription factors responsible for the Cys proteases, which led to an abundance of Cys proteases in ‘Valencia’ cells. To maintain a relatively balanced intercellular level of proteases, the activities of the inhibitors that can directly control Cys protease activity are also induced in the cells. The inhibition ratio of Cys proteases revealed the overproduction of Cys proteases in ‘Valencia’ sweet orange leaves. Yu and Killiny (2018) reported the presence of proteases and endoglucanase in the saliva of D. *citri*, potentially linked with the ability of the insects to degrade/inhibit citrus defense proteins. We hypothesized that the overaccumulation of cysteine proteases causes degradation of defense proteins and/or induces PCD and are linked to the enhanced susceptibility of ‘Valencia’ trees to *Ca*Las. SPs appear to be widespread in the xylem sap of different species (Buhtz et al., 2004). SPs have various functions in plant cells, including functions in the pathogen response (Tornero et al., 1997; Jordá et al., 1999). Specific citrus SPs were detected, suggesting that protease enzymatic activity is finely tuned during *Ca*Las infection (Franco et al., 2020). The overexpression of XSP1 in finger lime suggests that there is specific mechanism underlying the defense against *Ca*Las infection in finger lime tissues. Since the role of several genes reported in this study have not been elucidated before, further comprehensive studies are needed to understand in detail their role in the finger lime HLB defense pathways.

## Conclusions

To date, there are no effective practical strategies to mitigate citrus greening disease (HLB). Understanding the mechanisms against *Ca*Las can contribute to the development of effective approaches for combatting HLB. This report provides insights into different mechanisms of HLB tolerance in finger lime, an HLB tolerant citrus species. Finger lime trees protect themselves from *Ca*Las infection and disease symptoms through the control of ROS-overproduction by enhancing redox control factors such as glutathione-S-transferases (GSTs), catalase, thioredoxin and growth hormones to mitigate HLB symptoms. Primary recognition of ROS leads to stronger activation of defense responses against *Ca*Las infection. The over-accumulation of Cys proteases and their inhibitors in ‘Valencia’ linked with the degradation of citrus defense proteins such as the cysteine rich proteins. Finger lime did not exhibit any imbalance in the expression of the Cys proteases and their inhibitors. Several defense-related-factors were upregulated in the finger lime such as R-proteins, Cys-rich secretory proteins or PR proteins and hormone signaling-related genes. Additionally, we observed that ‘Valencia’ sweet orange phloem constitutively produced more callose and expressed more phloem proteins than finger lime following infection. Taken together, this study provides evidence that HLB can be managed by mitigating ROS and control of the over-accumulation of cysteine protease related genes that induce cell death (**Figure 9**).

## Data availability statement

The datasets presented in this study can be found in online repositories. The names of the repository/repositories and accession number(s) can be found below: https://www.ncbi.nlm.nih.gov/, PRJNA755969.

## Author Contributions

KW, LM, DS and MD - wrote the manuscript. LM - gene expression analysis. KW - transcriptome data analysis. DS – Microscopy. SW – callose density estimation. LM and WQ – RNA extraction. LM - statistical analysis. AL and JG – resources. MD – designed the study, obtained funding, and supervised the project. All authors contributed to the article and approved the submitted version.

## Funding

This work was supported by the Florida State legislative funding for the UF/IFAS Citrus Initiative.

## Acknowledgments

We thank E. Nielsen, C. Hardy, H Rubio, J. Thomson and K. Plant for aiding in various aspects of the work. Microscopy work was carried out at the Microscopy Core Facility of the Citrus Research and Education Center. We thank Novogene Corporation Inc. for library construction and RNA sequencing.

## Conflict of interest

The authors declare that the research was conducted in the absence of any commercial or financial relationships that could be construed as a potential conflict of interest.

## Publisher’s note

All claims expressed in this article are solely those of the authors and do not necessarily represent those of their affiliated organizations, or those of the publisher, the editors and the reviewers. Any product that may be evaluated in this article, or claim that may be made by its manufacturer, is not guaranteed or endorsed by the publisher.
