# Supplementary material for: Insights into the mechanism of Huanglongbing tolerance in the Australian finger lime (Citrus australasica)
Source: Front Plant Sci. 2022 Oct 21;13:1019295. doi: 10.3389/fpls.2022.1019295 (PMC9634478; doi:10.3389/fpls.2022.1019295)
Supplement: Supplementary file 2 [file DataSheet_1.pdf]

Supplementary Material

Insights Into the Mechanism of Huanglongbing (HLB) Tolerance in the Australian Finger Lime (*Citrus australasica*)

Kyle C. Weber[1†], Lamiaa M. Mahmoud[1,2†], Daniel Stanton[1], Stacy Welker[1], Wenming Qiu[3], Jude W. Grosser[1], Amit Levy[1] and Manjul Dutt[1*]

[1] Citrus Research and Education Center, University of Florida, Lake Alfred, FL 33850, USA.

[2] Pomology Department, Faculty of Agriculture, Mansoura University, 35516, Mansoura, Egypt

[3] Institute of Fruit and Tea, Hubei Academy of Agricultural Sciences, Wuhan, 430064, China

Correspondence: Manjul Dutt manjul@ufl.edu

[†]These authors contributed equally to this work.

Table S1. Sampling times for *Ca*Las diagnosis.

Sampling	Month
3 months	March
6 months	June
9 months	September
12 months	December
15 months	March
18 months	June
21 months	September
24 months	December

Table S2. TAQMAN based primer sequences used to amplify a 87-bp fragment of the *Ca*Las rplJ/rplL ribosomal protein gene

Primer	Sequence (5’ to 3’)
CQUL-F	TGGAGGTGTAAAAGTTGCCAAA
CQUL-R	CCAACGAAAAGATCAGATATTCCTCTA
CQUL-Probe	ATCGTCTCGTCAAGATTGCTATCCGTGATACTAG

Table S3. List of the primer sequences used in SYBR Green based qPCR assay.

Gene	Symbol	Forward and reverse primer sequences (5' to 3')
orange1.1g007239m	RLK8	GAGATTTCAGCTCTCCCTCTTC<br>GTAACCCTCGTTGGCTTCTT
orange1.1g041433m	RLK10-1	GTGGAAAGCAAGGAGGAAGA<br>GAGGTGGAGGAGAAAGAAGAAC
orange1.1g041917m	RLK10-2	GGTCACAGCCTCCTTACTTATAC<br>CTTCCTGAACGCACAACAATC
orange1.1g037707m	RLK10-3	CTGCTCTCTACTCTTCGGTCTA<br>AATCACCACGGCAGAGAAAG
orange1.1g021682m	RLK16	AATGGTGAAGGAATGGAGTTTATTG<br>TGGACACAGAGCAAAGCTAC
orange1.1g040682m	RLK18	AGATGTGTTTAGCTTTGGAGTATTG<br>CAAGCATGACCAAGAAGGTTAAA
orange1.1g017211m	RLK25	CAGCCTAAGGTACGAGGAATAC<br>TGATGATACAGTGGCGATTACA
orange1.1g017150m	RLK25	AGAGCTGTCAACGGAAGATTG<br>AGAAATCTGGCACCCTTCTTATC
orange1.1g006125m	RLK34	GGTGCCACAGAACCATCTAT<br>CCACACTGGCTAGAACGATAA
orange1.1g031237m	*CsCAP1*	GACTACGACATGAAGGGTTCAG<br>GAGTGTAATGGCCGCACATA
orange1.1g043403m	*CsCAP2*	TGGGTGAATGAGAAAGCTGATTA

		CGCCAAACCACCTGAGTATAG
orange1.1g037670m	*CsCAP3*	TACGGTGCCAACCAGTTATG<br>TTTGGCGCGCATGAATTATC
orange1.1g034999m	*CsLCR69*	CTACTTGTGCTGCTGCTTCT<br>AGCACAGTTGCTCTTACTCAC
orange1.1g028661m	Cysteine proteinases superfamily protein transcription factors	GTCAAGGAGCAGCAAAGGA<br>CTGCTATAGCCTTCTGTCCAATTA
orange1.1g032006m		AAGGAGACCCTGGAGCTAAA<br>GCACAAACTGAAGGGCAATAAA
orange1.1g019112m		GTGGAAGGGATCACTCAGATTAC<br>GCATCCATTGTTGTCTGTTGAG
orange1.1g046431m	Phloem protein 2-B1	CACTGTCCCATCATCTCCATTT<br>CATCAACGGTATGTCCCATCTT
orange1.1g042543m	Phloem protein 2-B13	CTAGTGCAAGAGACTGGAAAGAA<br>CGTAGAGAAAGCCAGTTCCAATA
orange1.1g045590m	Phloem protein 2-B15	CGAGAGAGCTTTCGGATTAGAC<br>CTTTCTTGCCAACGCAAATAAAC

Table S4. List of the primer sequences used in SYBR Green based real-time PCR assay for RNAseq validation.

Gene	Forward and reverse primer sequences (5' to 3')
orange1.1g025919m	GAGGGATATGGGACAAACACAG<br>GGCACCATCTTGGGTCATTA
orange1.1g004503m	TAGGGTGGCTGCATACAAAG<br>ACGTTCACACCGTCATGAATA
orange1.1g000943m	CTGATCTCAACACCAGACCTAAC<br>AGCAGCAATGATGGGTGAATA
orange1.1g041335m	CCGACTGGTTTAGCCATCAA<br>AGGAACAGCACAATAAGCAAAC
orange1.1g044721m	ACG GTC GGT CAC ACT TTA TTA G<br>TTC CCA AAT TCT GAA GGG AAG A
orange1.1g040540m	ACTCATTCTCGGGCCATATTC<br>AGAGGATTCTGTCGTCAAGTTATT
orange1.1g020713m	GTTGAAGCCCAAGAAGACAAATC<br>TCCTCCATCGGTATCCATCTT
orange1.1g008415m	GGAAGAAGGCGATGATCATGTA<br>CTCCAGCGTGAACCAGAAA
orange1.1g019200m	TCCCTCTGGACTCGTGATATT<br>GACCAAGAGGACTGTATGGAAC
orange1.1g020892m	AGAGATCCGAGAGGTTCACTTA<br>TTTGCCACCAGAACCTGTAATA
orange1.1g041918m	AAGAGGAGTGCGGGATTTG

	AACATACCAGTCCCGATAACAC
orange1.1g027498m	CCTGAAGTCATGGGAGCATT<br>GAGCCCTTTCCAGCATCTT
orange1.1g004923m	AGGGAGTTACCAAGCAAATAGG<br>GTGTTGATCCAGAACCACTGA
orange1.1g005031m	GATCACAAAGCTGCTCAATCAC<br>GTCCGGCAATGTAGGAAAGA
orange1.1g047288m	GCAGCTATTGCCAATCAAGAAA<br>TGGAGAAGTTGCCTGACATAAA
orange1.1g008038m	GCCACAGACACTGCTGATA<br>CGCACTGTTCATTCCCTCTTA

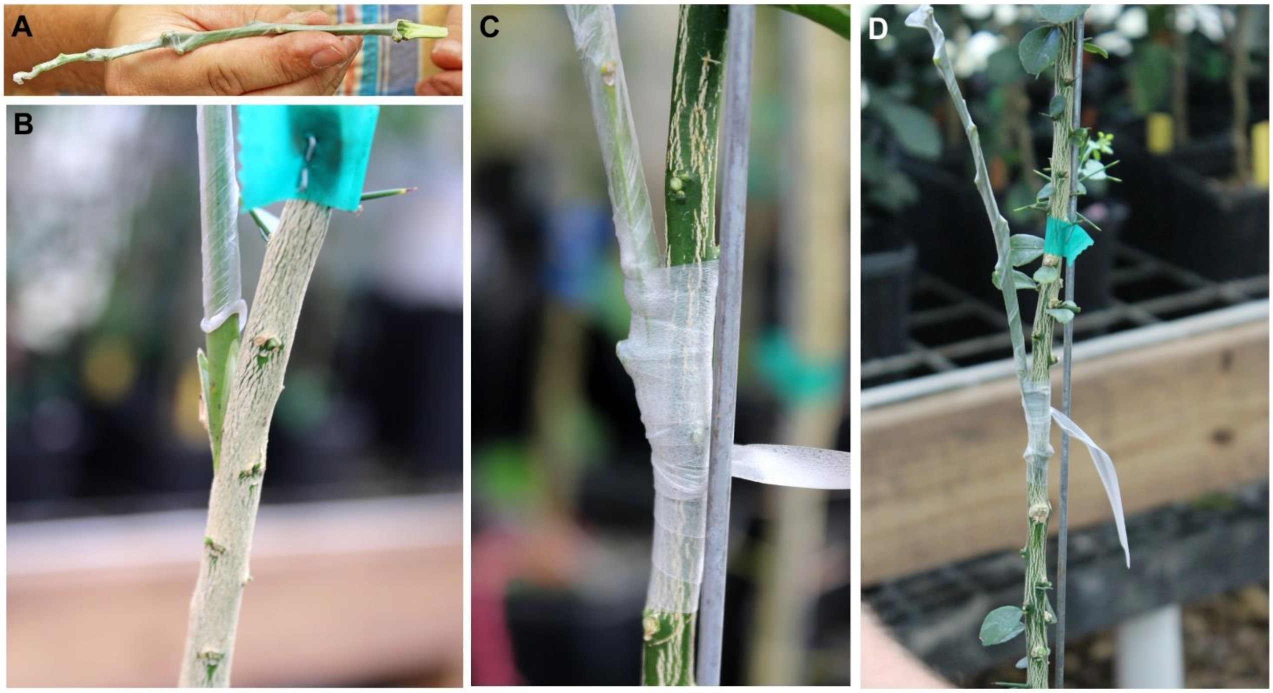

Figure S1. Wedge grafting in Finger Lime A) Parafilm wrapped HLB infected ‘Valencia’ budstick end cut into a "V" shaped wedge. B) A similar sized side cut was made into the cambial tissue of the Finger lime and the ‘Valencia’ budstick inserted into the cambial tissue. Care is taken to align cambia from both. C) Finger lime stock and ‘Valencia’ budstick tied together with 5/8-Inch thick, budding tape. D) Another view of successfully side grafted Finger Lime with infected ‘Valencia’ budstick.

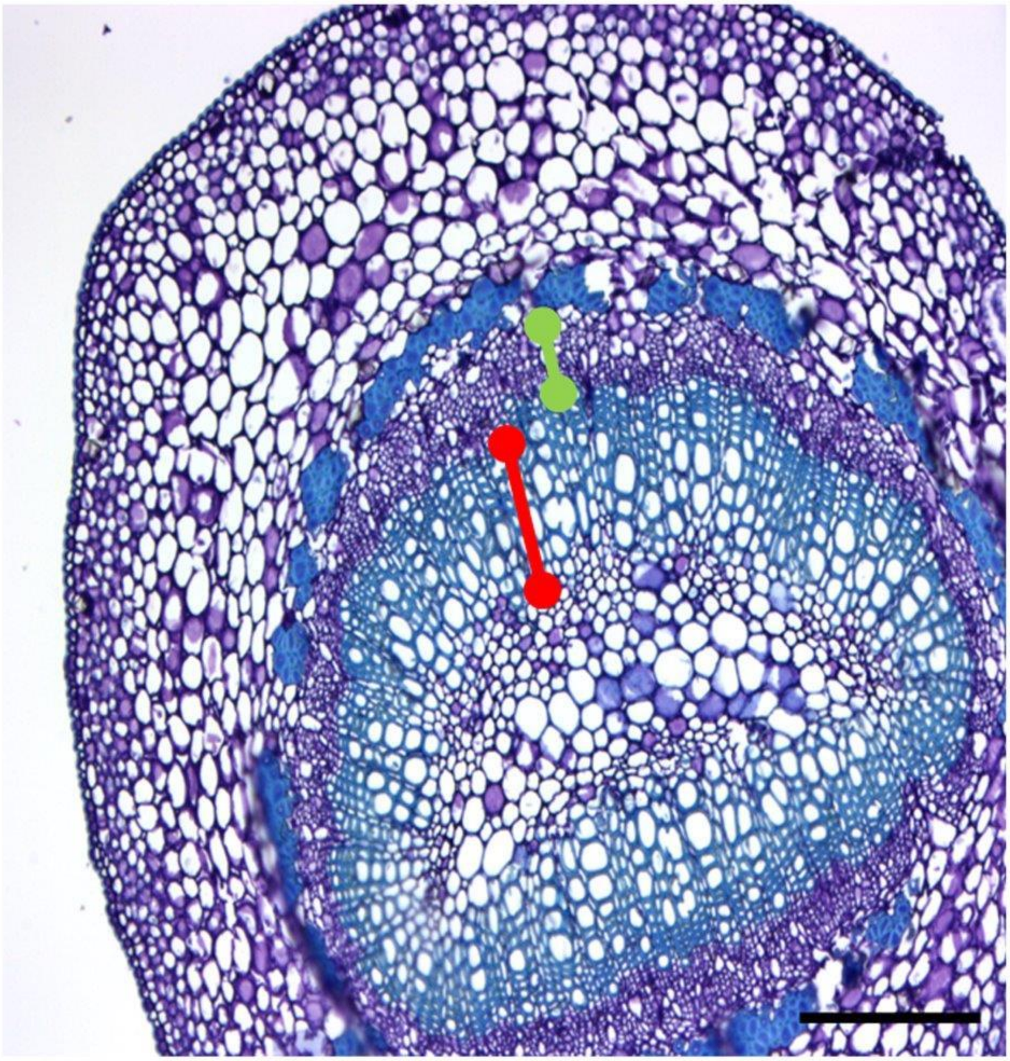

Figure S2. The image illustrates technique used to measure the phloem and xylem ring distance. Red line represents the xylem, and the green line represents the phloem.

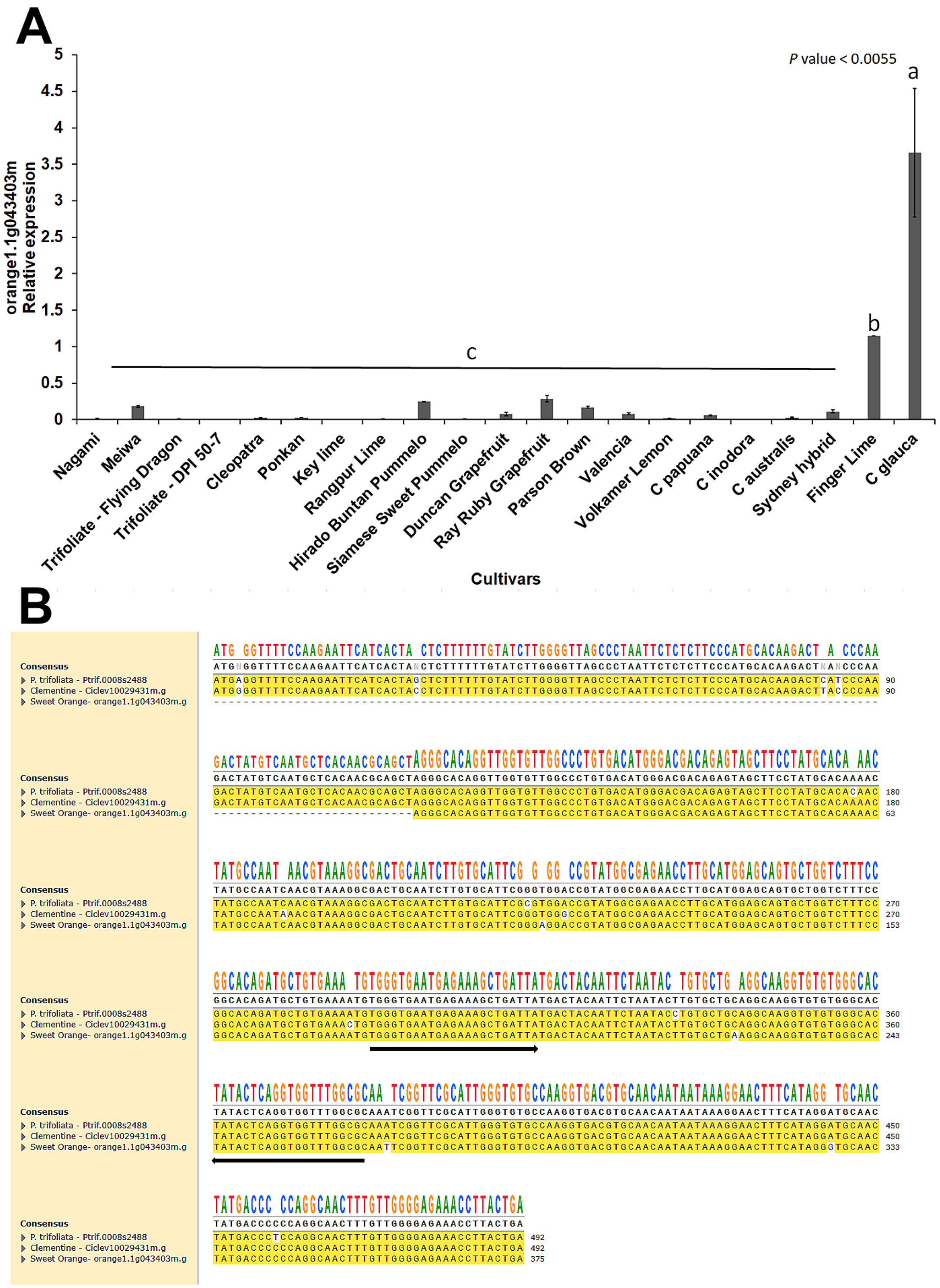

Figure S3. A) Relative transcript levels of the cysteine-rich secretory protein (orange1.1g043403m) calculated by qPCR and compared with the finger lime trees. The trees were growing in the field under similar environmental conditions. Data are means ± SE of three samples. **B)** Multiple sequence alignment of the cysteine-rich secretory protein (Sweet Orange - orange1.1g043403m) and related sequences available in the Phytozome database (Clementine - Ciclev10029431m.g and *Poncirus trifoliata* - Ptrif.0008s2488). Arrow indicates the forward and reverse qPCR primer locations.

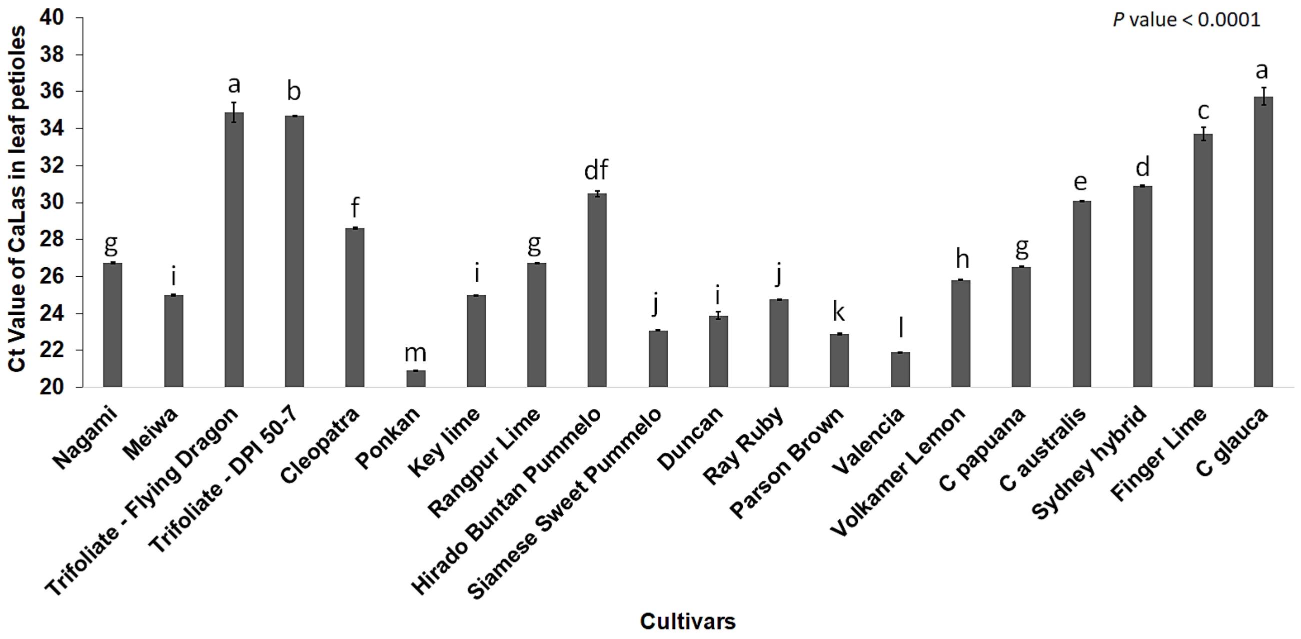

Figure S4. Detection of *Ca*Las in leaf tissues of different citrus varieties by qPCR. The trees were growing in the field under similar environmental conditions. Data are means ± SE of three samples.
